# Global burden on drug use disorders from 1990 to 2021 and projections to 2046

**DOI:** 10.3389/fpubh.2025.1550518

**Published:** 2025-07-28

**Authors:** Chen Dongying, Sun Yanyan, Li Xiaowu, Yin Zongyi

**Affiliations:** ^1^Department of Anesthesia, Shenzhen University General Hospital, Shenzhen University, Shenzhen, China; ^2^Department of Hepatobiliary Surgery, Shenzhen University General Hospital, Shenzhen University, Shenzhen, China; ^3^Guangdong Provincial Key Laboratory of Regional Immunity and Diseases & Carson International Cancer, Shenzhen University, Shenzhen, China; ^4^Shenzhen University Clinical Medical Academy Center, Shenzhen University, Shenzhen, China

**Keywords:** Global Burden of Disease (GBD), drug use disorders, substance use disorders, incidence, prevalence, death, disability-adjusted life years

## Abstract

**Background:**

Drug use disorders (DUDs) continue to pose a heavy burden on individuals, families, and societies. Despite extensive research, there remains a paucity of comprehensive reports on the spatiotemporal distribution, driving factors, and future trends of DUDs at global, regional, and national levels. This study aims to address this gap by investigating these critical aspects of the DUDs epidemic.

**Methods and findings:**

Annual data on DUDs-related burden were collected from the Global Burden of Diseases, injuries, and risk factors Study (GBD) 2021. Age-period-cohort (APC) analysis and estimated annual percentage change were used to evaluate the spatiotemporal trend of burden. Decomposition analysis was used to identify the temporal and population-specific variations in the burden. The slope index of inequality and the concentration index were utilized to summarize health inequality of the burden. Frontier analysis was performed to evaluate the relationship between the burden of DUDs and socio-demographic development. The Nordpred model and Bayesian age-period-cohort (BAPC) model were introduced to forecast the burden. In 2021, the global prevalence of DUDs reached 53,115,936 (95% UI: 46,999,805–60,949,054), marking a 35.50% increase since 1990 and is projected to continue rising over the next 25 years. The increment in incidence, deaths, and disability-adjusted life years (DALYs) was 35.50%, 122.22%, and 74.65%, respectively. Despite the declining trends in global rates of incidence, prevalence, and DALYs, mortality still shows an upward trend, increasing from 1.26 to 1.65 per 100,000. Opioid and cocaine use disorders were the primary contributors to the overall increase in DUDs DALYs (82.07 and 59.57%, respectively). Population growth was the primary driver of the increase in DUDs DALYs (35.31%). A higher burden was observed in males and populations aged 15–39 years. Health inequality and insufficient healthcare performance regarding DUDs remain prominent issues in both high and low socio-demographic index (SDI) regions.

**Conclusions:**

This study underscores the persistent and evolving nature of DUDs. Future research should focus on understanding the complex interplay between age and gender disparities, socioeconomic development, drug policies, and DUDs burden to inform more effective global strategies.

## Introduction

Drug use disorders (DUDs) constitute a significant global health challenge, imposing a substantial burden on individuals, families, and societies worldwide (1–3). According to the World Drug Report 2023, substance use affected more than 296 million individuals globally in 2021 (4). The report also highlighted a significant upward trend in DUDs, with a 45% surge observed over the recent decade, and only 20% of those grappling with DUDs receiving pharmacological interventions. Moreover, harmful use of drugs is responsible for 494,000 deaths annually and the loss of 30.9 million healthy years of life due to premature death and disability (5). Furthermore, the disparity in treatment availability across different geographical areas has continued to expand, exacerbating the challenges faced by affected populations in certain regions (5).

Despite extensive research, several challenges persist in understanding the full scope of DUDs: (1) the importance of DUDs as a public health issue among young people is not reflected in, and remains unaddressed by, the allocated resources for an extended period (5). (2) DUDs generally receive limited research investment and political support compared to other non-communicable diseases (4). (3) The spatiotemporal distribution of DUDs presents another challenge, as patterns of drug use vary significantly across regions and change over time (1, 3). (4) The complex relationship between DUDs and socioeconomic development remains incompletely understood, with some studies suggesting a paradoxical increase in drug use with economic growth in certain contexts (4). These knowledge gaps underscore the need for comprehensive, global studies that can capture the nuanced trends of DUDs across different countries and regions.

This study aims to address these challenges by analyzing data from the Global Burden of Disease (GBD) study 2021 (6–8). The GBD study allows for a comparison of cause-specific disease burden over time and by country through the standardization of data management and methods. By examining trends in incidence, prevalence, deaths, and disability-adjusted life years (DALYs) for various drug use disorders from 1990 to 2021, we seek to provide a comprehensive overview of the changing landscape of DUDs over the past three decades. This analysis will contribute valuable insights into the global, regional, and national patterns for informing evidence-based policies and interventions to address this global health issue effectively.

## Methods

### Data sources

For the study, we extracted data pertaining to DUDs burden and population statistics from the Global Health Data Exchange (GHDx) query tool (https://vizhub.healthdata.org/gbd-results/). This resource provided us with detailed information on DUDs-related burden, encompassing incidence, prevalence, mortality, and DALYs. The data were disaggregated by various demographic factors, including, sex, and geographical location for the period 1990–2021. Socio-demographic index (SDI) was collected from (https://ghdx.healthdata.org/record/global-burden-disease-study-2021-gbd-2021-socio-demographic-index-sdi-1950%E2%80%932021). Healthcare Access and Quality (HAQ) index was collected from.

### Cause definition and classification

In GBD 2021, the DUDs were defined based on the Diagnostic and Statistical Manual of Mental Disorders (DSM-IV-TR) or the International Classification of Diseases (ICD-10) diagnostic criteria, including opioid use disorders, cocaine use disorders, cannabis use disorders, amphetamine use disorders, and other DUDs (6–8). Other DUDs included hallucinogen dependence, inhalant or solvent dependence, sedative dependence, tranquilizer dependence, and other medicines, drugs, substance dependence (6–8).

### Measures of burden

The key metrics used to assess DUDs burden included prevalence, incidence, mortality, and DALYs. The estimation process for these metrics incorporated sophisticated statistical modeling techniques, tailored to capture the complex nature of DUDs across various demographic and geographic dimensions (6–8). For the estimation of prevalence, incidence, and years lived with disability (YLDs), the study leveraged the Bayesian meta-regression tool DisMod-MR 2.1 (6). The study reported these burden measures in two formats: absolute numbers and age-standardized rates per 100,000 population. For age standardization, the World Health Organization's world population standard age structure was employed as the reference population (9).

### Spatial-temporal trend analysis

To elucidate the temporal trends in the burden of DUDs, we employed several sophisticated statistical approaches: (1) estimated annual percentage change (EAPC): we calculated the EAPC for age-standardized rates and absolute numbers of incidence, mortality, prevalence, and DALYs associated with DUDs over the period 1990–2021 (10). The EAPC was subsequently computed as: EAPC = 100× (exp(β) – 1). (2) Age-period-cohort (APC) analysis: we implemented an APC analysis to disentangle the effects of age, period, and birth cohort on DUDs trends (11).

### Decomposition analysis

To elucidate the complex dynamics underlying the temporal and population-specific variations in the burden of DUDs, we implemented a rigorous decomposition analysis. This analytical approach allows us to quantify the relative contributions of three primary factors driving changes in the DUDs burden: population growth; population aging; and epidemiologic changes (12, 13).

### Health care access and quality

To address the potential non-linear relationship between the HAQ Index and DALYs, we employed a sophisticated statistical approach. The HAQ Index was modeled as a restricted cubic spline function, while simultaneously controlling for SDI (13, 14). Knots for the cubic function were strategically placed at each quartile to capture the nuanced relationships between these variables. We examined the relationship between age-standardized DALY rates for DUDs in 2021 and the corresponding HAQ Index values from 2019 (13).

### Health inequality analysis

We utilized the slope index of inequality (to assess absolute inequality) and the concentration index (to assess relative inequality) to summarize health inequality. A key strength of both the sophisticated metrics lies in their population-weighted approach to calculation. This methodology ensures that the resulting single numerical value encapsulates inequality across all subgroups while accounting for variations in population size (15, 16).

### Frontier analysis

To evaluate the relationship between the burden of DUDs and socio-demographic development, we employed a frontier analysis as a quantitative methodology (13). The DALYs frontier delineates the minimum DALYs that could theoretically be attained for each country or territory given its SDI value. To account for uncertainty in our estimates, we utilized 100 bootstrapped samples of the data, randomly sampling with replacement from all countries and territories across all years. We computed the mean DUDs DALYs at each SDI value from these bootstrapped samples. Subsequently, we developed a locally weighted regression model with a local polynomial degree of 1 and a span of 0.3 to generate a smoothed frontier.

### Forecasting analysis

To evaluate the trends of DUDs over the next 25 years, we employed two sophisticated models: the Nordpred model and the Bayesian age-period-cohort (BAPC) model (17, 18). These models account for three types of time-varying phenomena: age effects, period effects, and cohort effects. To validate the stability of the prediction results, we further applied the BAPC model integrated with nested Laplace approximations to perform a sensitivity analysis (17, 19).

### Statistical analysis

Uncertainty intervals (UIs) were used to describe the point estimates of uncertainty from model specification, stochastic variation, and measurement bias. The point estimate is defined by the mean of the draws, while the 95% UIs are represented by the 2.5th and 97.5th percentiles of ranked estimates from the draws. All statistical analyses and visualization of results were conducted using the R software (Version 4.3.3; https://www.R-project.org/), and the two-tailed *P* value < 0.05 was considered statistically significant.

## Results

### Global burden overview of DUDs

The global landscape of DUDs has undergone significant changes over the past three decades. In 2021, the global incidence of DUDs reached 13,609,362.38 (95% UI: 11,625,287.78–15,667,184.2), marking a 35.50% increase since 1990. Despite this absolute increase, the age-standardized incidence rate (ASIR) showed a slight decline from 184.31 (95% UI: 156.91–211.67) per 100,000 in 1990 to 169.39 (95% UI: 145.14–195.01) in 2021, with an EAPC of −0.28 (95% UI: −0.27 to −0.27). Among specific substances, cannabis and opioids dominated the ASIR, with 46.77 (95% UI: 35.25–61.17) and 24.54 (95% UI: 20.74–29.48) per 100,000, respectively. The prevalence of DUDs also increased by 34.06% from 1990 to 2021, reaching 53,115,936.38 (95% UI: 46,999,805.19–60,949,054.28) cases in 2021. However, the ASPR decreased from 709.15 (95% UI: 618.81–824.54) to 663.8 (95% UI: 584.52–766.14) per 100,000 over this period. The disease burden, as measured by DALYs, increased substantially by 74.65% from 1990 to 2021, reaching 162,061.67 (95% UI: 110,807.96–213,561.19) DALYs in 2021. The age-standardized DALY rate (ASDR) rose from 166.44 (95% UI: 132.55–198.4) to 190.97 (95% UI: 156.11–222.79) per 100,000. Deaths attributed to DUDs also saw a dramatic increase of 122.22% from 1990 to 2021, with 137,277.92 (95% UI: 129,268.62–146,181.36) deaths in 2021. The age-standardized mortality rate (ASMR) increased from 1.26 (95% UI: 1.17–1.37) to 1.65 (95% UI: 1.55–1.75) per 100,000 (Table 1, Supplementary Tables S1–S3).

**Table 1:** Number, crude rate, ASPR for overall DUDs in 2021 and percentage change from 1990.

**Factors**	**Prevalence number**		**Prevalence rate**		**Overall**		**Opioid**		**Cocaine**		**Amphetamine**		**Cannabis**		**Other drug**	
**Location**	**Number**	**EAPC**	**Crude rate**	**EAPC**	**ASR**	**EAPC**	**ASR**	**EAPC**	**ASR**	**EAPC**	**ASR**	**EAPC**	**ASR**	**EAPC**	**ASR**	**EAPC**
Global	53,115,936.38 (46,999,805.19–60,949,054.28)	0.95 (1.04–0.88)	673.09 (595.59–772.35)	−0.32 (−0.23 to −0.38)	663.8 (584.52–766.14)	−0.21 (−0.18 to −0.24)	198.49 (173.42–227.22)	0.81 (0.91–0.73)	50.63 (39.74–63.79)	−0.25 (−0.11 to −0.42)	115.99 (84.63–153.55)	−1.53 (−1.54 to −1.55)	286.23 (222.58–384.31)	−0.14 (−0.12 to −0.09)	18.17 (14.82 to 22.12)	−0.09 (−0.03 to −0.12)
East Asia	8,012,070.41 (6,895,063.34–9,433,061.45)	−1.21 (−1.17 to −1.23)	544.01 (468.17–640.5)	−1.81 (−1.77 to −1.84)	589.83 (494.67–703.92)	−1.02 (−1.1 to −0.98)	94.72 (77.62–112.73)	−2.21 (−2.34 to −2.13)	6.13 (4.18–8.59)	−0.72 (−0.79 to −0.71)	269.62 (195.03–362.11)	−1.49 (−1.6 to −1.4)	205.9 (154.31–284.04)	0.69 (0.71–0.76)	15.84 (12.5–20.06)	−1.1 (−1.16 to −1)
Oceania	98,706.44 (72,689.38–133,720.37)	2.47 (2.59–2.39)	708.71 (521.91–960.11)	0.01 (0.12 to −0.08)	672.72 (503.93–893.96)	0.02 (0.05 to −0.03)	68.86 (56.55–82.59)	0.09 (0.14–0.07)	2.3 (1.35–3.42)	−0.23 (−0.35 to −0.2)	136.57 (92.19–189.06)	−0.03 (−0.01 to −0.05)	455.6 (298.54–672.23)	0.02 (0.09 to −0.01)	11.67 (9.17–14.79)	0 (0.05 to −0.07)
Central Asia	563,159.84 (480,004.35–667,119.29)	1.2 (1.29–1.09)	587.8 (501–696.3)	0.14 (0.24–0.04)	574.49 (483.62–687.87)	0.14 (0.15–0.12)	213.65 (185.05–247.5)	0.08 (0.27 to −0.02)	22.51 (16.26–30)	0.33 (0.49–0.2)	127.6 (91.05–172.64)	0.28 (0.35–0.18)	198.04 (129.47–302.45)	0.11 (0.13–0.11)	15.04 (11.97–18.87)	0.1 (0.18–0.01)
Australasia	530,216.75 (478,541.18–594,230.42)	0.35 (0.37–0.3)	1,712.51 (1,545.6–1,919.26)	−1.01 (−0.99 to −1.06)	1,819.35 (1,632.77–2,054.7)	−0.66 (-0.65 to−0.68)	284.21 (259.19–311.84)	0.31 (0.33–0.31)	225.8 (164.31–314.59)	0.22 (0.16–0.25)	513.42 (371.55–682.47)	0.11 (0.16–0.12)	718.11 (599.67–860.36)	−1.71 (−1.75 to −1.7)	102.72 (87.2–118.37)	0.84 (0.94–0.75)
Western Europe	4,519,736.55 (4,106,574.42–5,029,749.49)	0.13 (0.23–0.06)	1,033.34 (938.88–1,149.95)	−0.29 (−0.19 to −0.36)	1,201.17 (1,081.17–1,351.18)	0.21 (0.3–0.16)	237.54 (213.94–263.02)	0.83 (0.88–0.77)	138.33 (100.6–190.17)	0.27 (0.28–0.22)	187.82 (135.17–251.47)	0.39 (0.43–0.35)	598.62 (494.1–743.63)	−0.11 (−0.01 to −0.14)	49.29 (41.46–57.13)	0.89 (1.02–0.73)
High-income North America	12,918,133.53 (11,781,271.57–14,224,633.86)	2.6 (2.77–2.43)	3,489.74 (3,182.63–3,842.68)	1.69 (1.86–1.52)	3,668.01 (3,323.49–4,067.36)	1.98 (2.14–1.82)	1,890.26 (1,659.84–2,156.24)	5.82 (5.92–5.75)	479.97 (379.72–592.54)	0.5 (0.76–0.26)	334.25 (249.54–432.38)	1.29 (1.78–0.87)	973.88 (752.87–1,275.29)	−0.16 (−0.19 to −0.16)	76.38 (64.09–90.25)	2.3 (2.46–2.17)
Southeast Asia	3,872,468.94 (3,139,748.22–4,776,982.49)	1.29 (1.37–1.22)	554.55 (449.62–684.08)	−0.03 (0.05 to −0.1)	524.3 (424.34–649.16)	0.03 (0.04–0.02)	54.3 (45.42–63.78)	0.1 (0.18–0.07)	1.99 (1.22–2.92)	−0.44 (−0.46 to −0.4)	189.63 (130.4–262.39)	−0.15 (−0.1 to −0.15)	269.94 (194.27–381.28)	0.14 (0.14–0.1)	10.24 (8.04–13.11)	0.2 (0.22–0.13)
Southern Latin America	565,190.58 (505,940.75–640,496.67)	1.42 (1.5–1.35)	834.91 (747.39–946.16)	0.4 (0.48–0.33)	815.88 (730.49–926.72)	0.36 (0.44–0.3)	110.9 (86.57–136.22)	−0.26 (−0.23 to −0.33)	250.22 (188.83–332.21)	0.21 (0.26–0.15)	72.35 (49.93–99.31)	0.05 (0.1 to −0.02)	369.24 (325.22–426.04)	0.79 (1.06–0.63)	17.59 (13.28–23.51)	−0.03 (−0.01 to −0.07)
Andean Latin America	339,995.28 (286,939.55–407,831.52)	1.98 (2.04–1.95)	514.11 (433.88–616.68)	0.18 (0.23–0.14)	480.27 (406.37–576.63)	0.02 (0.02–0.02)	99.23 (77.66–122.43)	0.06 (0.1–0.03)	64.08 (46.11–90.87)	0.2 (0.22–0.15)	68.86 (47.6–95.21)	0.19 (0.21–0.23)	236.64 (172.76–327.74)	−0.09 (−0.14 to −0.03)	12.96 (10.02–16.73)	0.11 (0.09–0.13)
South Asia	7,844,210.59 (6,513,450.04–9,819,055.31)	2.22 (2.33–2.12)	424.8 (352.73–531.75)	0.51 (0.61–0.41)	391.33 (327.33–483.68)	0.09 (0.16–0.03)	105.86 (87.16–126.68)	0.4 (0.44–0.38)	3.18 (2.19–4.42)	−0.04 (0.06 to −0.16)	11.62 (8.13–15.85)	0.17 (0.19–0.19)	260.4 (198.16–346.85)	−0.05 (−0.02 to −0.1)	10.92 (8.67–13.69)	0.59 (0.59–0.53)
Central Sub-Saharan Africa	403,026.92 (309,609.12–542,840.8)	3.24 (3.27–3.23)	294.33 (226.11–396.44)	0.25 (0.28–0.23)	306.32 (246.24–397.68)	0.09 (0.11–0.09)	69.81 (56.86–83.82)	0.29 (0.34–0.23)	7.95 (5.88–10.39)	0.35 (0.39–0.26)	38.7 (26.71–54.28)	0.05 (0.06–0.05)	180.66 (124.03–266.94)	0.01 (0.02–0.01)	9.69 (7.75–12.09)	0.09 (0.17–0)
Eastern Sub-Saharan Africa	1,411,567.64 (1,067,353.55–1,896,497.99)	2.91 (2.9–2.92)	331.28 (250.49–445.09)	0.28 (0.26–0.29)	325.17 (257.73–422.17)	−0.03 (−0.03–0.02)	60.32 (49.34–71.65)	0.01 (0.06 to −0.06)	5.17 (4.11–6.43)	0.45 (0.55–0.27)	36.3 (25.41–51.02)	0 (0.01 to −0.02)	216.64 (152.35–307.84)	−0.06 (−0.14–0.01)	7.2 (5.67–9.18)	0.26 (0.4–0.17)
High–income Asia Pacific	1,203,526.58 (1,028,637.41–1,489,142.38)	−0.64 (−0.55 to −0.73)	648.99 (554.68–803)	−0.85 (−0.77 to −0.95)	781.29 (644.13–995.33)	−0.07 (−0.08 to −0.09)	90.22 (71.08–109.56)	−0.16 (−0.13 to −0.19)	101.46 (72.92–139.47)	−0.11 (−0.15 to −0.16)	110.32 (76.1–153.56)	−0.09 (−0.08 to −0.12)	466.09 (328.88–669.92)	−0.04 (−0.06 to −0.06)	16.87 (12.49–22.85)	−0.14 (−0.13 to −0.16)
Central Europe	644,902.46 (569,858.99–745,988.94)	−0.59 (−0.4 to −0.7)	559.5 (494.4–647.2)	−0.32 (−0.14 to −0.44)	662.66 (571.49–776.46)	0.16 (0.28–0.06)	89.23 (77.04–104.1)	0.64 (0.84–0.54)	35.91 (25.34–49.44)	0.14 (0.18–0.07)	181.72 (129.43–246.17)	0.51 (0.63–0.45)	342.62 (264.57–440.97)	−0.13 (0.03 to −0.29)	16.52 (13.06–21.12)	0.39 (0.47–0.35)
Eastern Europe	1,935,373.74 (1,732,723.49–2,177,484.65)	−0.41 (−0.32 to −0.48)	936.05 (838.04–1,053.15)	−0.12 (−0.03 to −0.18)	1,041.24 (908.44–1,198.5)	0.25 (0.29–0.23)	431.53 (379.31–493.25)	0.28 (0.35–0.19)	48.53 (36.71–62.33)	0.53 (0.61–0.4)	203.57 (153.65–264.85)	0.23 (0.38–0.09)	336.82 (230.68–483.34)	0.19 (0.15–0.19)	29.41 (23.88–35.65)	0.28 (0.32–0.26)
Southern Sub–Saharan Africa	541,071.64 (455,101.09–657,468.16)	1.47 (1.42–1.54)	673.78 (566.72–818.73)	0.08 (0.03–0.15)	639.35 (539.97–771.88)	−0.15 (−0.27 to −0.02)	134.96 (114.3–157.31)	−1.29 (−1.28 to −1.31)	89.26 (67.95–114.91)	0.43 (0.49–0.35)	113.54 (80.78–152.22)	−0.4 (−0.41 to −0.41)	291.16 (205.32–413.03)	0.46 (0.32–0.6)	12.85 (10.24–16.05)	0.26 (0.3–0.21)
Caribbean	355,322.75 (277,254.27–455,909.08)	0.78 (0.81–0.71)	748.7 (584.2–960.64)	−0.18 (−0.15 to −0.25)	733.78 (568.3–946.83)	0.03 (−0.04–0.06)	88.28 (70.52–108.35)	−0.67 (−0.68 to −0.64)	106.38 (77.47–147.92)	0.08 (0.12–0.02)	51.23 (35.87–70.57)	0.11 (0.19–0.11)	476.43 (315.55–696.65)	0.16 (0.05–0.18)	14.34 (11.57–17.95)	−0.32 (−0.26 to −0.34)
Tropical Latin America	2,099,771.44 (1,788,693.83–2,510,048.37)	1.14 (1.34–0.97)	922.88 (786.15–1,103.2)	−0.16 (0.04 to −0.32)	888.21 (750–1,066.61)	−0.06 (0.04 to −0.16)	90.82 (71.67–113.42)	−0.31 (−0.27 to −0.27)	195.3 (144.63–256.82)	1.26 (1.32–1.04)	174.84 (121.64–239.06)	−0.16 (−0.1 to −0.22)	421.92 (314.68–564.89)	−0.45 (−0.31 to −0.61)	10.86 (8.44–13.92)	0.82 (0.95–0.71)
Central Latin America	1,420,513.92 (1,237,211.37–1,635,360)	1.77 (1.86–1.62)	561.46 (489.01–646.38)	0.37 (0.45–0.22)	529.59 (460.9–609.7)	0.25 (0.28–0.17)	87.65 (69.37–107.42)	−0.21 (−0.18 to −0.23)	106.31 (76.67–148.96)	−0.17 (−0.16 to −0.2)	50.38 (35.05–69.23)	−0.01 (0.03 to −0.07)	274.83 (220.77–346.77)	0.69 (0.87–0.52)	12.15 (9.46–15.57)	−0.29 (−0.34 to −0.24)
North Africa and Middle East	2,771,196.58 (2,422,239.49–3,187,679.94)	2.65 (2.77–2.55)	444.81 (388.8–511.67)	0.65 (0.77–0.56)	422.68 (369.99–485.49)	0.35 (0.4–0.33)	222.34 (190.3–258.56)	0.37 (0.52–0.24)	17.26 (12.39–23.08)	−0.06 (0.01 to −0.14)	37.14 (26.45–50.55)	0.31 (0.39–0.2)	133.59 (93.62–187.44)	0.41 (0.39–0.42)	13.53 (10.54–17.3)	0.28 (0.31–0.21)
Western Sub–Saharan Africa	1,065,773.81 (875,503.32–1,332,478.03)	3.22 (3.29–3.18)	217.58 (178.74–272.03)	0.16 (0.23–0.13)	236.64 (199.54–285.92)	0.03 (0.07–0.02)	61.75 (49.74–74.58)	−0.05 (0.01 to −0.1)	7.8 (6.52–9.37)	0.86 (1.03–0.73)	33.73 (23.57–47.81)	−0.12 (−0.12 to −0.12)	124.85 (91.97–173.12)	0.04 (0.13–0.01)	8.81 (7.03–11.16)	0.52 (0.7–0.4)

ASR, age-standardized rate per 100,000 residents; EAPC, estimated annual percent change (%); data in () indicates the uncertainty interval, it reflects the certainty of an estimate based on data availability, studies size and consistency across data sources.

**Figure 1:**
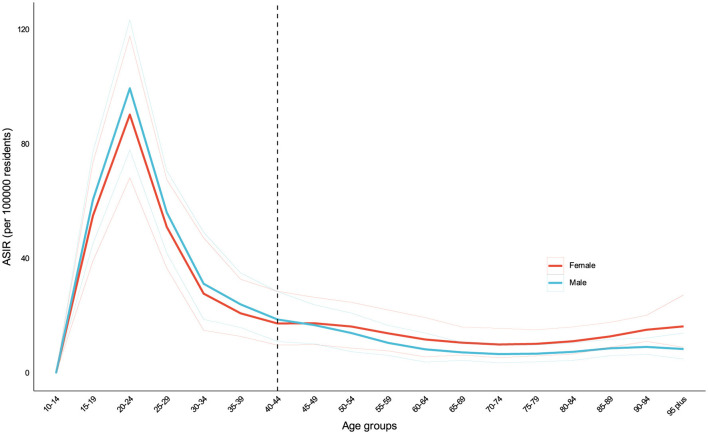
ASIR of DUDs by sex in 2021. Age and gender disparities were evident in the incidence of DUDs. The highest incidence rates were observed in young adults aged 15–39 years, peaking at approximately 88 per 100,000 in the 20–24 age group. A gender differential was noted, with males showing higher incidence rates before age 40, while females exhibited higher rates after age 40.

All specific drug categories exhibited positive EAPCs in death rates over this period. Notably, opioid use disorders were the only category to show a positive estimated annual percentage change (EAPC) in ASPR [0.81 (95% UI: 0.73–0.91)]. Cannabis and opioid use disorders demonstrated increasing trends in ASDR, with EAPCs of 0.45 (95% UI: 0.29–0.49) and 0.23 (95% UI: 0.18–0.26), respectively. Age and gender disparities were evident in the incidence of DUDs. The highest ASIR were observed in young adults aged 15–39 years, peaking at ~88 per 100,000 in the 20–24 age group. A gender differential was noted, with males showing higher ASIR before age 40, while females exhibited higher rates after age 40 (Figure 1, Supplementary Figures S1, S2).

### The burden of DUDs in 2021 and temporal trends

**Figure 2:**
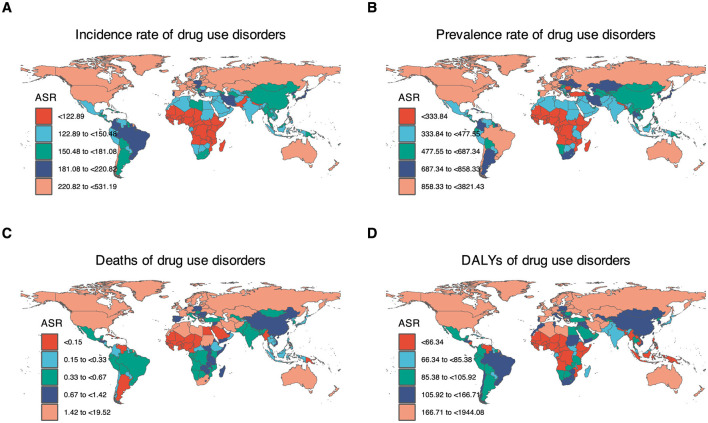
Age-standardized rates of DUDs-related burden in 2021 by countries or territories. **(A)** ASIR of drug use disorders. **(B)** ASPR of drug use disorders. **(C)** ASPR of drug use disorders. **(D)** ASDR of drug use disorders. In 2021, the highest burden of DUDs was concentrated in High-income North America, Australasia, and Europe. Countries near the equator have a relatively lower burden of DUDs.

In 2021, the highest burden of DUDs was concentrated in High-income North America, Australasia, and Western Europe. At the national level, USA, Australia, Canada, and Estonia generally exhibited higher ASPR of DUDs compared to other countries or territories. The USA demonstrated the highest prevalence with 12,146,953.91 cases (95% UI: 11,024,582.17–13,461,043.9), followed by China with 7,680,058.66 cases (95% UI: 6,602,083.42–9,057,281.31), and India with 6,366,009.45 cases (95% UI: 5,297,783.08–7,997,066.8). In terms of incidence, China led with 2,451,314 new cases (95% UI: 2,046,472.04–2,907,370.53), followed by India (2,047,672.59; 95% UI: 1,706,130.84–2,396,268.76), USA (1,583,449.64; 95% UI: 1,384,480.18–1,793,912.27), and Brazil (411,752.6; 95% UI: 350,243.05–474,141.24). High-income countries, particularly USA, Australia, and Canada, tended to have higher ASIR, ASMR, and ASDR (Figure 2, Supplementary Figures S3, S4).

Over the 32-year study period, Estonia, USA, and Lithuania experienced the most significant increases in ASIR. Conversely, China, Switzerland, and Italy showed notable declines. Regarding changes in absolute incidence cases, Qatar, the United Arab Emirates, Equatorial Guinea, and Jordan demonstrated more than a three fold increase over the past three decades. Interestingly, a gender disparity was observed across different SDI levels. In high SDI countries, ASIR were higher among males, while low and middle SDI countries showed an opposite trend with higher rates among females (Supplementary Figures S5, S6).

### Drivers factors of DUDs burden

To elucidate the factors shaping the epidemiology of DUDs over the past three decades, we conducted a decomposition analysis of incidence, prevalence, deaths, and DALYs. This analysis considered three primary drivers: population growth, aging, and epidemiologic changes, the latter represented by age- and population-standardized morbidity and mortality rates.

**Figure 3:**
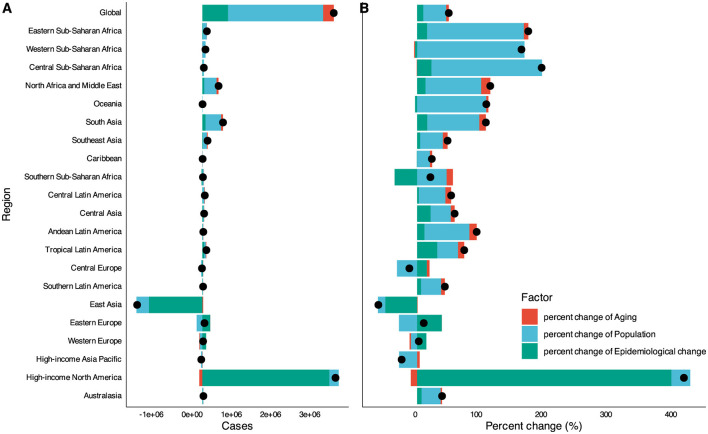
Drivers factors of DUDs DALYs from 1990 to 2021. **(A)** The number of changes contributed by all three factors. **(B)** The percent of change contributed by all three factors. Globally, there was a significant increase in DUDs DALYs, with the most pronounced increases drove by population growth. The exception is High-income North America, where epidemiological change is the main driving factor. East Asia had a significant decrease in DUDs DALYs contributed by epidemiological change. The black dot represents the overall value of change contributed by all three components.

Globally, there was a significant increase in DUDs DALYs, with the most pronounced increases observed in High-income North America and South Asia. Conversely, East Asia exhibited a notable decline. Our analysis revealed that population growth was the primary contributor to the increased burden of DUDs DALYs between 1990 and 2021, accounting for 35.31% of the increase, followed by epidemiologic changes at 9.48%. The impact of population growth on overall DALYs was most evident in Sub-Saharan Africa (165.30%), North Africa and Middle East (85.77%), and South Asia (80.23%). Aging contributed most significantly to overall DALYs in North Africa and Middle East (14.10%), South Asia (10.04%), and Andean Latin America (11.24%). In low and middle SDI-quintiles, population growth was the primary driver of increased DUDs DALYs. Epidemiologic changes, reflecting underlying shifts in age- and population-adjusted DUDs burden over the 32-year period, showed a global increase. This increase was particularly pronounced in High-income North America. Notably, East Asia and Southern Sub-Saharan Africa were the only regions where epidemiologic changes led to a decrease in DUDs DALYs. Regarding mortality, the most significant increases in DUDs deaths were observed in High-income Asia Pacific (96.32%), Eastern Europe (150.85%), and Western Europe (181.56%) (Figure 3 and Supplementary Figures S7–S9).

Country-level decomposition analysis revealed substantial heterogeneity in demographic and epidemiologic trends. In most high-income countries, epidemiologic changes and population growth were the major drivers of changes in DUDs DALYs. In contrast, aging and population growth were the primary drivers in most developing countries.

### Decomposition analysis by causes of DUDs

**Figure 4:**
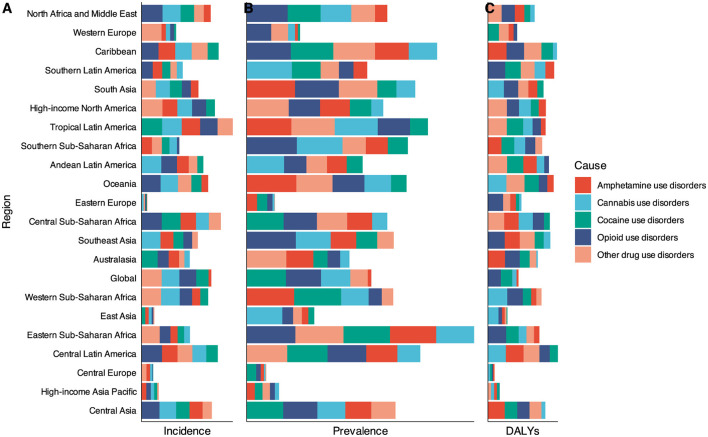
Changes in DUDs burden according to the four causes from 1990 to 2021. **(A)** Changes in DUDs incidence cases. **(B)** Changes in DUDs prevalence cases. **(C)** Changes in DUDs DALYs. Opioid and cocaine use disorders were the primary contributors to the overall increase in DUDs burden. There were significant regional differences in the overall increase in DUDs burden contributed by these five types of drugs.

To elucidate the differential contributions of specific DUDs to the overall burden, we conducted decomposition analyses for five major categories: opioid use disorders, cocaine use disorders, amphetamine use disorders, cannabis use disorders, and other drug use disorders. Globally, opioid and cocaine use disorders were the primary contributors to the overall increase in DUDs DALYs, accounting for 82.07 and 59.57% of the increase, respectively. The impact of opioid use disorders on the change in overall DALYs was particularly pronounced in Southeast Asia (59.49%), Southern Latin America (38.34%), and Eastern Sub-Saharan Africa (183.50%). Amphetamine use disorders emerged as a significant driver of change in the overall DUDs burden in specific regions, contributing 54.46 and 56.95% of the change in DUDs DALYs in Australasia and Central Asia, respectively. Notably, the burden of DUDs in High-income Asia Pacific, Central Europe, East Asia, and Eastern Europe was comparatively lower than in other regions. Cannabis use disorders were identified as the leading driver of change in DUDs DALYs, although its relative contribution varied substantially across geographical regions. Its impact was particularly high, exceeding 50% in Southern Latin America, East Asia, and Oceania. From 1990 to 2021, Cannabis use disorders, followed by opioid use disorders and cocaine use disorders, were the primary drivers of increased DALYs globally and across all SDI-quintiles (Figure 4).

### The burden of DUDs and sociodemographic development

**Figure 5:**
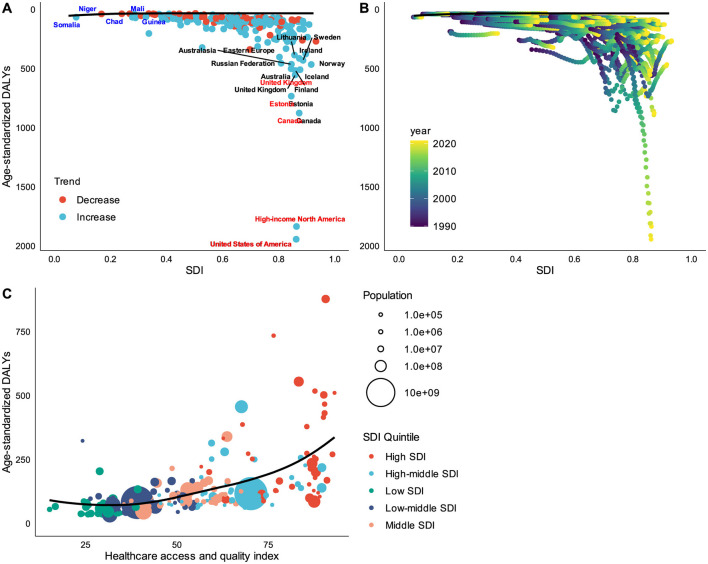
Frontier analysis based on SDI and ASDR from 1990 to 2021. **(A)** The effective difference from the frontier for each country or territory by single year (2021 vs. 1990). **(B)** The effective difference from the frontier for each country or territory by all years (from 1990 to 2021). **(C)** The relationship between healthcare access and quality index and ASDR. The frontier line delineated the countries or territories with lowest ASDR (optimal performers) given their SDI. Higher SDI countries had larger gap between their country's observed and potentially achievable DALYs; this gap could be potentially reduced or eliminated based on the country or territory's sociodemographic resources. High HAQ scores also showing relatively higher ASDR.

To elucidate the potential for improvement in DUDs DALY rates relative to a country's development status, we conducted a frontier analysis. This analysis examined the relationship between age-standardized DALY rates and the SDI using data from 1990 to 2021. The effective difference from the frontier for each country or territory was calculated using the 2021 DALYs and SDI values. We found that: variability in ASDR was observed across all SDI levels from 1990 to 2021, with this variability appearing to increase at higher SDI values; high-income countries (e.g., USA, Canada, UK, Australia) exhibited higher ASDR despite their high SDI, indicating significant challenges with DUDs in developed countries; countries with low SDI (e.g., Niger, Somalia, Chad) demonstrated lower DALY rates, potentially due to factors such as reduced drug availability, under-reporting, or cultural differences in attitudes toward drug use; the United States stood out with an exceptionally high DALY rate despite its high SDI, suggesting a particularly severe drug use problem (Figures 5A, B).

To further investigate the distribution of the DUDs burden in relation to countries' health system performance, we examined the relationship between burden measures and the HAQ index. This analysis revealed: a positive relationship between HAQ and ASDR, with countries exhibiting high HAQ scores also showing relatively higher ASDR; after accounting for regional confounds and controlling for SDI, a near-linear positive relationship between ASDR and HAQ was also observed (Figure 5C).

### The burden of DUDs and health inequality

**Figure 6:**
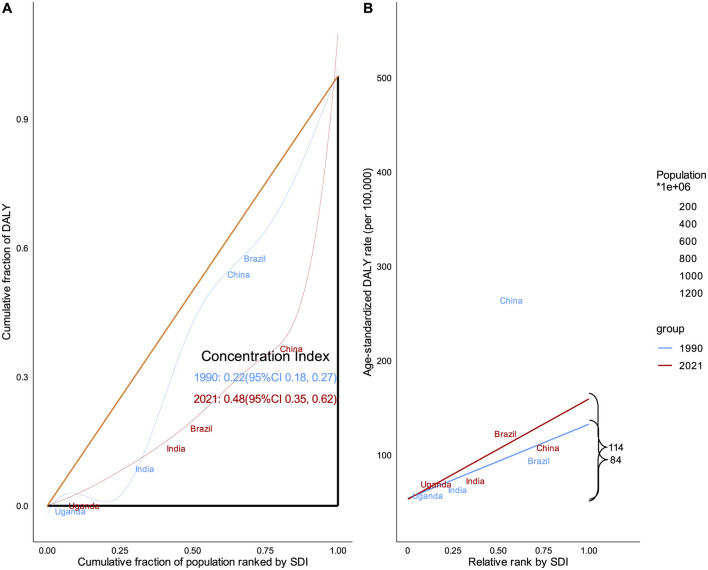
Health inequality analysis based on SDI and ASDR from 1990 to 2021. **(A)** Relative inequality analysis. **(B)** Absolute inequality analysis. Global health inequalities in the burden of DUDs have significantly worsened over the past three decades. The burden of DUDs is disproportionately concentrated in countries with higher socioeconomic development.

To identifying health inequalities and their drivers in achieving health equity, we conducted an in-depth analysis of relative and absolute health inequalities in the burden of DUDs. Our findings reveal that global health inequalities in the burden of DUDs have significantly worsened over the past three decades, the concentration index was 0.22 (95% CI 0.18, 0.27) in 1990 and 0.48 (95% CI 0.35, 0.62) in 2021 (*P* < 0.01). The burden of DUDs is disproportionately concentrated in countries with higher socioeconomic development. The USA emerges as a striking outlier, exhibiting exceptionally high DALY rates at both time points examined. Moreover, the country demonstrated a marked increase in its ASDR in 2021 compared to 1990. China and Brazil, despite their large populations, display relatively low ASDR. These countries have experienced increases in ASDR from 1990 to 2021, signaling a growing health inequality in these populous countries. In contrast, India and Uganda, representative of low/middle SDI countries, exhibits relatively low ASDR with minimal change observed between 1990 and 2021, indicating the health inequalities situation in this country has not significantly changed (Figure 6).

### Prediction of DUDs burden in the next 25 years

**Figure 7:**
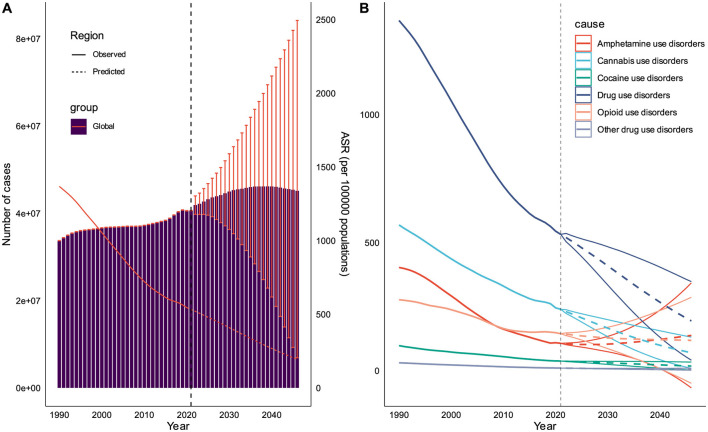
Prediction of DUDs burden in the next 20 years. **(A)** Prediction for the overall prevalence cases and ASPR of DUDs at global level. **(B)** Prediction for ASPR of DUDs subtypes.

Forecasting the future burden of DUDs can provides essential insights for policymakers and healthcare administrators to effectively plan and allocate resources. Our predictive analysis for the next two decades reveals several key trends: the overall number of DUDs prevalence is projected to continue its upward trajectory over the next 25 years, albeit at a decelerated rate; a notable shift is anticipated in the landscape of specific DUDs, with opioid use disorders predicted to surpass cannabis use disorders in ASPR by ~2030; DUDs-related incidence cases, prevalence cases and DALYs would increase to 10,176,246, 45,105,497, and 18,822,146, respectively; these increased cases in some countries represent a substantial multiplication of the corresponding number observed in 2021; in contrast to the absolute number increases, the ASIR, ASPR, ASDR are projected to decline to approximately half of their 2021 levels; a divergent trend is anticipated in High-income North America, particularly in USA, where both absolute numbers and age-standardized rates are expected to increase, contrary to the global trend (Figure 7 and Supplementary Figures S10, S11).

## Discussion

This comprehensive analysis of GBD 2021 reveals significant trends and patterns in the global burden of DUDs. It revealed that the absolute number of DUDs exceeded 53 million people in 2021, and is projected to continue rising over the next 25 years. Despite the declining trends in global ASPR, ASIR, and ASDR, the ASMR still shows an upward trend, even without including mortality data for cannabis use disorders. Higher burden was observed in males, 15–39 years old populations. Population growth was the primary contributor to the increased burden of DUDs DALYs, accounting for 35.31%. Health inequality and insufficient healthcare performance regarding the burden of DUDs remains a prominent issue, both in high SDI and low SDI regions.

The global incidence and prevalence of DUDs have shown substantial increases in absolute numbers over the past three decades, although age-standardized rates have declined. This paradoxical trend can be largely attributed to population growth and changes in age structure, particularly in low and middle SDI countries (4). Besides, many low and middle SDI countries have strengthened drug prevention education over the past decade. For instance, according to the United Nations Office on Drugs and Crime (UNODC), countries like China, Kenya, and Nigeria have introduced drug prevention education into school curricula (5). However, the significant rise in ASMR associated with DUDs is particularly concerning. The reason may be that, despite potential decreases in overall incidence rates, many regions still lack adequate treatment resources (5, 20). Prevention efforts may have helped reduce new cases in some regions, but the increased potency of drugs and the rise of polydrug use have made existing cases more severe.

The age and gender disparities observed in DUDs incidence highlight the need for targeted interventions. Our findings indicated that DUDs were still very serious among young adults. This age group is particularly vulnerable to DUDs due to a combination of neurobiological, psychological, and social factors (21–24). The earlier the use of psychoactive drugs, the greater the lifelong risk of DUDs (23). This age group may suffer deprivation, poverty, homelessness, famine, gender-based discrimination and frequent displacement (20, 23). As a result, they can develop various mental and physical health issues. Thus, reducing contact with drugs and better treatment services for DUDs should be provided promptly to accurately identify and meet the needs of such people (21, 22). The gender differential, with males showing higher rates before age 40 and females after, suggests the need for gender-specific approaches in both prevention and treatment programs (22). Males were more likely to receive higher doses of psychotropic drugs and suffer from DUDs and drug dependence before age 40 (21–23). The 2021 World Drug Report indicates that men are about twice as likely as women to use cannabis, cocaine, or amphetamines (4). After age 40, females were more prone to mental disorders and dependent on psychotropic drugs compared to males (5, 22). Additionally, there are more obstacles for females in accessing medication, leading to insufficient medication treatment (4, 5). They may endure more social stigmatization, fear legal sanctions, and possibly even lose custody of their children. Therefore, more practical and effective strategies for women should also be developed and implemented to alleviate or even relieve these gender-specific burdens.

Geographical variations in DUDs burden reveal significant disparities between high-income and low/middle-income countries. The concentration of high prevalence rates in North America, Australasia, and Western Europe may reflect differences in drug availability, societal attitudes, and reporting practices (4, 19). However, the rapid increases observed in some developing countries, particularly in the Middle East and Africa, signal an urgent need for proactive measures in these regions. The higher prevalence of DUDs in high SDI countries can be attributed to several factors: (1) greater economic resources: higher disposable incomes may increase access to drugs. For instance, the USA, with its high SDI, has seen a significant, partly fueled by the widespread prescription of opioid painkillers (19, 20). (2) Advanced drug trafficking networks: developed countries often have more sophisticated drug distribution systems. The European Monitoring Center for Drugs and Drug Addiction reports that online drug markets on the dark web have grown significantly, with annual revenues estimated to be in the hundreds of millions of euros (25). (3) Cultural factors: some high SDI countries have more permissive attitudes toward recreational drug use. For example, the Netherlands' policy of tolerance toward cannabis has led to higher reported use rates compared to many other European countries (25, 26). Conversely, the rapid increase in DUDs in some developing countries, particularly in the Middle East and Africa, can be explained by: (1) demographic dividend: many developing countries have a large youth population, who are more susceptible to drug us (27). (2) Weak regulatory frameworks: many developing countries lack robust systems to control prescription drugs, leading to their misuse. For instance, tramadol abuse has become a significant problem in parts of Africa and the Middle East, with the UNODC reporting seizures increasing from 10 tons in 2010 to over 125 tons in 2017 (4, 5).

The decomposition analysis provides crucial insights into the drivers of DUDs epidemiology. While population growth emerges as the primary contributor to increased DUDs burden globally, the significant role of epidemiologic changes in certain regions, particularly High-income North America, suggests that factors beyond demographics are at play (4, 19). These may include changes in drug potency, shifts in drug use patterns, and variations in healthcare and policy responses. The dominance of cannabis and opioids in incidence rates reflects global patterns of drug availability and use. The increasing trend in opioid use disorder prevalence is especially alarming, given the high mortality risk associated with opioid use (4, 5, 20). This trend aligns with the ongoing opioid crisis in several countries, particularly in North America. The differential impact of specific drug categories across regions highlights the need for tailored approaches to drug policy and intervention. The dominant role of opioid and cocaine use disorders in driving the global increase in DUDs DALYs calls for intensified efforts in prevention, treatment, and harm reduction for these substances.

The relationship between DUDs burden and socio-economic development exhibits a complex pattern, defying simple correlations. While high SDI countries generally show higher prevalence rates of DUDs, the rapid increases observed in some lower SDI countries indicate that economic development alone may not lead to reduced drug use problems. Economic development can have contradictory effects on DUDs. While it may improve healthcare systems and prevention efforts, it can also increase disposable income and drug availability (5, 20). A study found that for every 10% increase in GDP per capita across 181 countries, there was an associated 4.3% increase in the prevalence of drug use (28). Implementing preemptive strategies may result in a relatively low official drug use prevalence (29, 30). Besides, decriminalizing personal drug use and investing heavily in treatment and harm reduction may be another successful policy (30).

We found that health inequality and insufficient healthcare performance regarding DUDs remains a prominent issue, both in high SDI and low SDI regions. In high SDI regions, despite abundant overall medical resources, DUDs patients may face social stigma and discrimination, leading to reluctance in seeking help or inability to access appropriate treatment (28). Simultaneously, healthcare systems might lack comprehensive intervention programs specifically tailored for DUDs or suffer from inadequate policy implementation. In contrast, low SDI regions may confront more fundamental challenges, such as a shortage of specialized medical professionals, limited financial resources, and underdeveloped healthcare infrastructure, all of which directly impact the accessibility and quality of DUDs-related services (4, 5, 28). To ameliorate this situation, multi-faceted strategies are necessary. For instance, performing comprehensive reforms to integrate DUDs prevention, treatment, and rehabilitation services into routine medical care, while enhancing the capacity of primary healthcare to manage DUDs (31, 32).

While acknowledging previous discussions on GBD limitations, it remains crucial to elucidate the specific constraints of this study (6, 7). Firstly, the GBD 2021 study defines DUDs based on DSM-IV-TR or ICD-10 criteria. The adoption of DSM-5 criteria could potentially alter DUDs estimates, as it introduces changes in diagnostic thresholds and criteria (33). Secondly, despite improvements in GBD 201′s modeling approach, the limited granularity of data from developing countries and regions may lead to underestimation of DUDs burden in these areas (34). Lastly, our study's predictions, based on GBD 2021 data, may lack precision due to the inherent lag in data reporting and collection. The rapidly evolving nature of drug use patterns, exemplified by the opioid crisis in North America or the rise of new psychoactive substances globally, means that even recent data may not fully capture current trends.

## Data Availability

The original contributions presented in the study are included in the article/supplementary material, further inquiries can be directed to the corresponding authors.
