# Supplementary material for: Global burden on drug use disorders from 1990 to 2021 and projections to 2046
Source: Front Public Health. 2025 Jul 28;13:1550518. doi: 10.3389/fpubh.2025.1550518 (PMC12336172; doi:10.3389/fpubh.2025.1550518)
Supplement: Supplementary file 1 [file Data_Sheet_1.pdf]

Global burden on drug use disorders from 1990 to 2021 and projections to 2046

Chen Dongying[4], Sun Yanyuan[4,*], Li Xiaowu[1,2,3,*], Yin Zongyi[1,2,3,*]

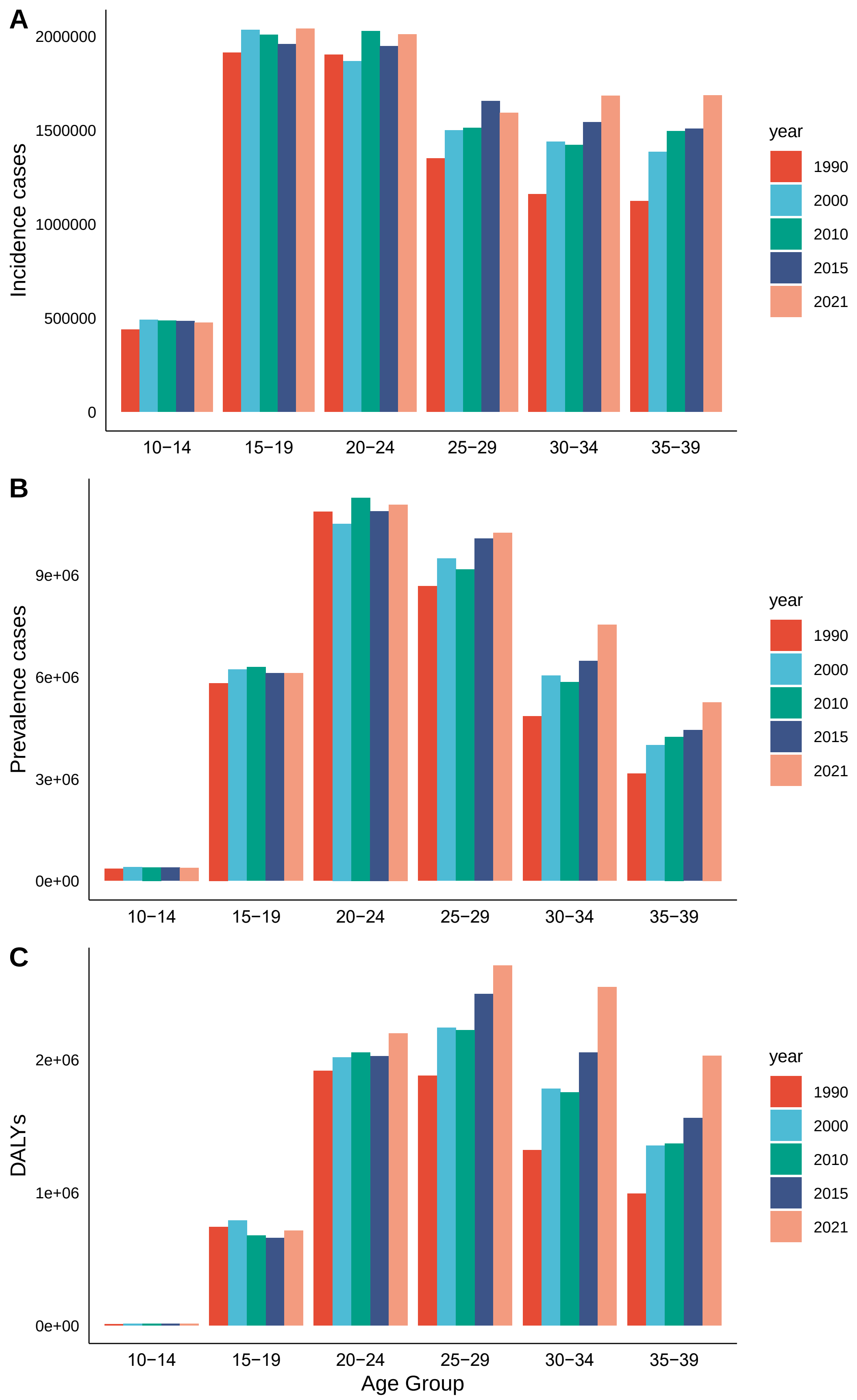

A
Incidence cases
0
500000
1000000
1500000
2000000
10−14
15−19
20−24
25−29
30−34
35−39
year
1990
2000
2010
2015
2021
B
Prevalence cases
0e+00
3e+06
6e+06
9e+06
10−14
15−19
20−24
25−29
30−34
35−39
year
1990
2000
2010
2015
2021
C
DALYs
0e+00
1e+06
2e+06
10−14
15−19
20−24
25−29
30−34
35−39
Age Group
year
1990
2000
2010
2015
2021

sFig. 1 Age distribution of DUDs burden from 1990 to 2021. A. Incidence cases. B. Prevalence cases. C. DALYs.

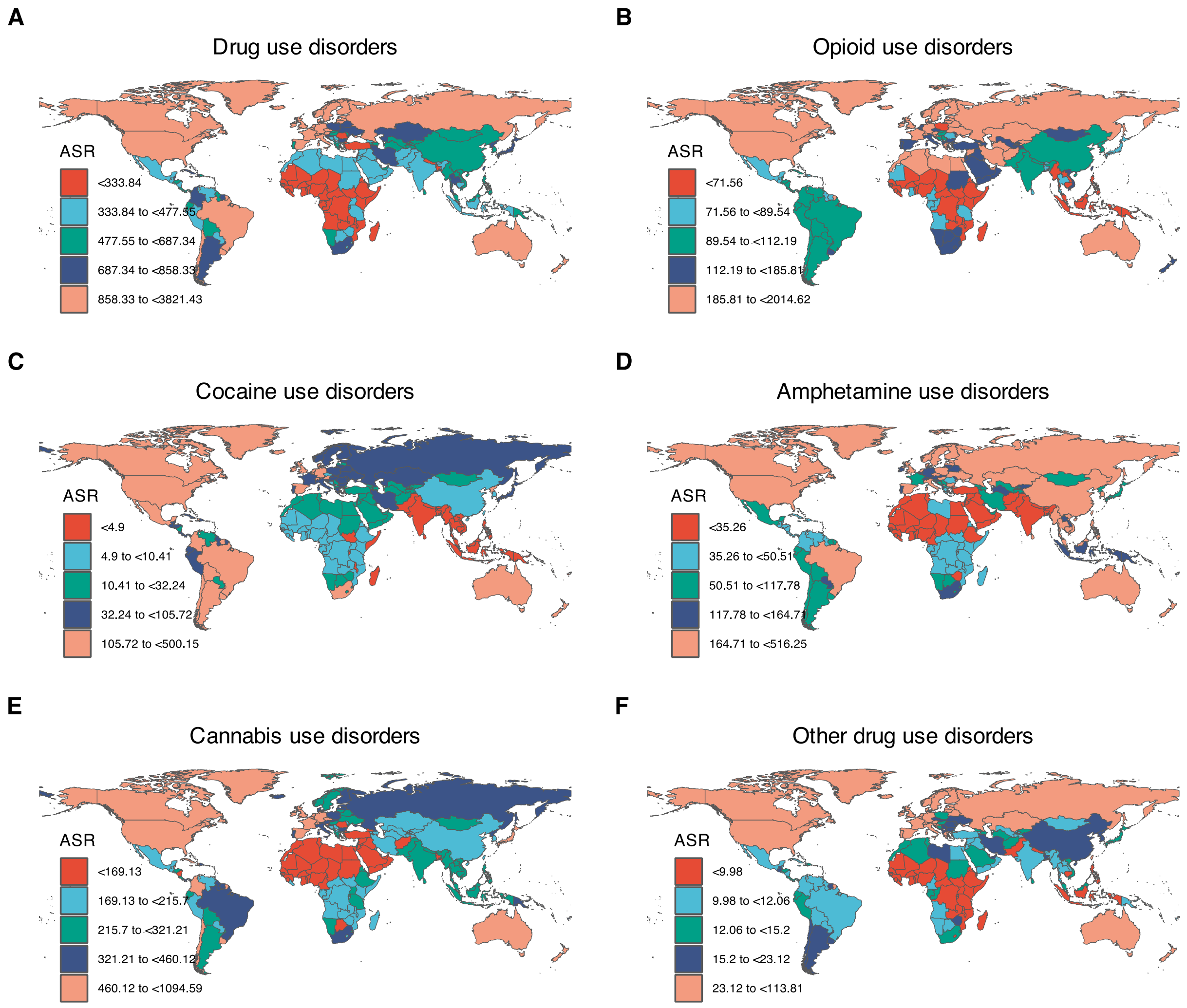

sFig. 2 Global distribution of ASPR for different types of DUDs in 2021.

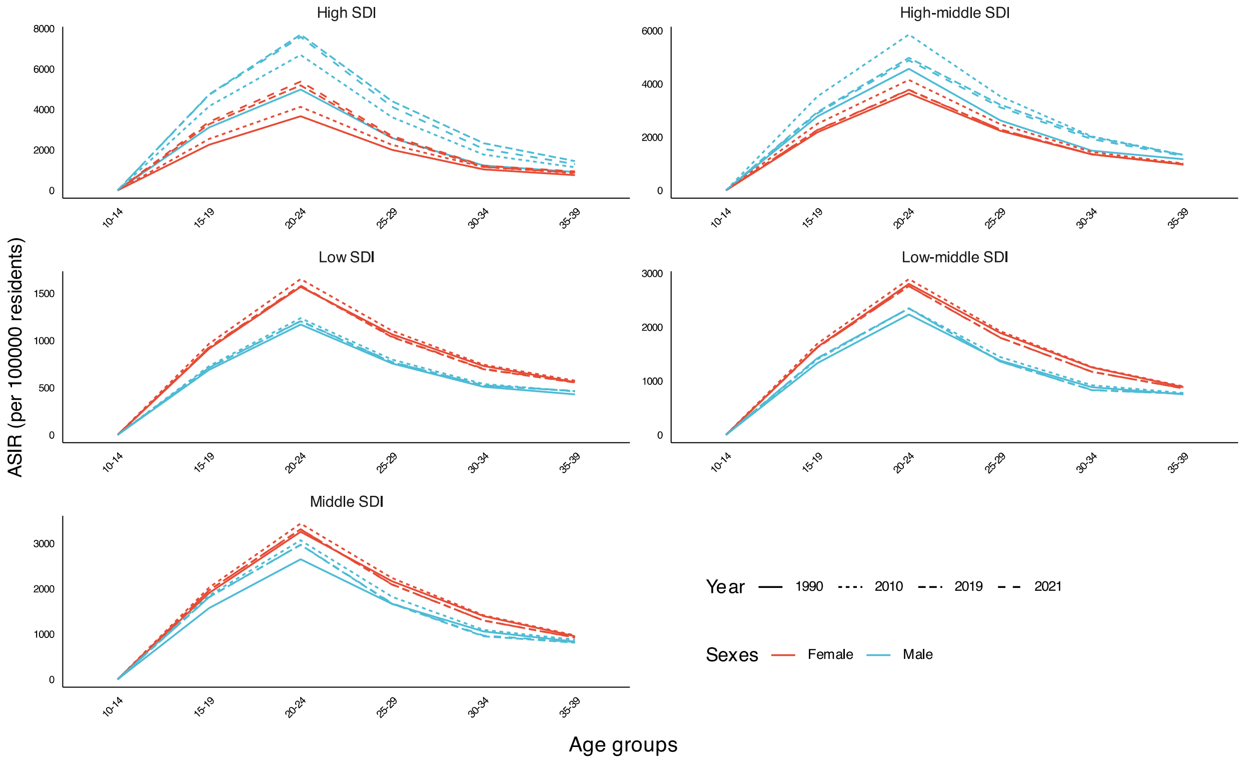

sFig. 3 ASIR of DUDs by SDI level, sex, and year (1990-2021)

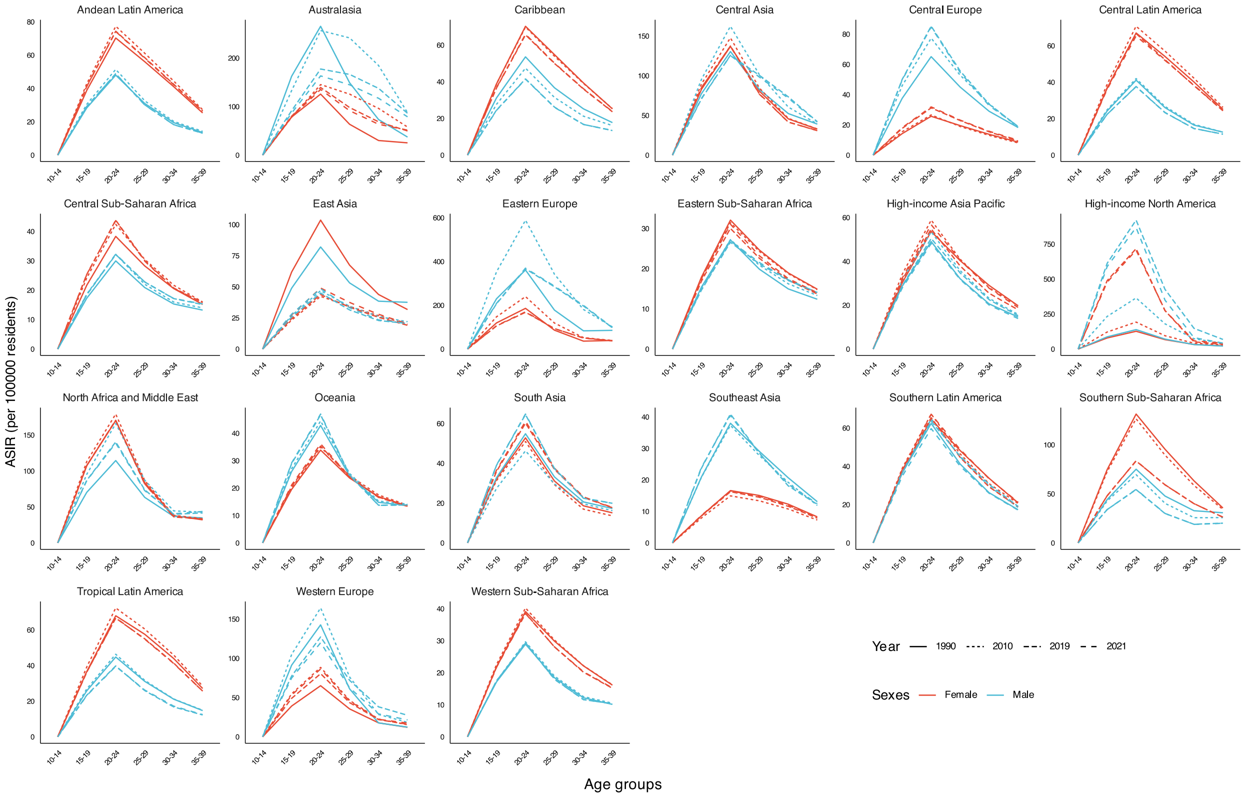

sFig. 4 ASIR of DUDs by regional level, sex, and year (1990-2021)

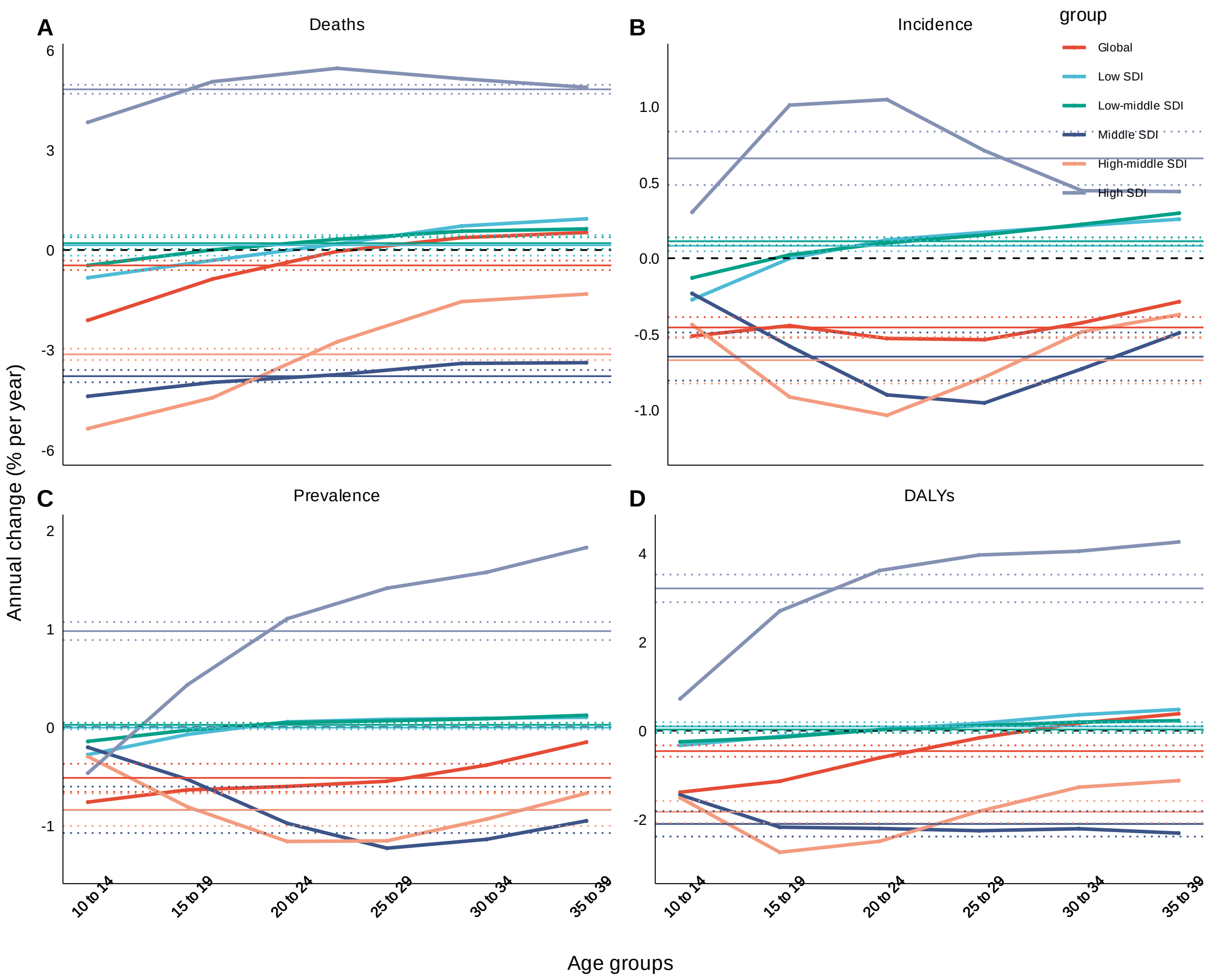

sFig. 5 Age-specific annual change of DUDs burden by SDI level, sex, and year (1990-2021)

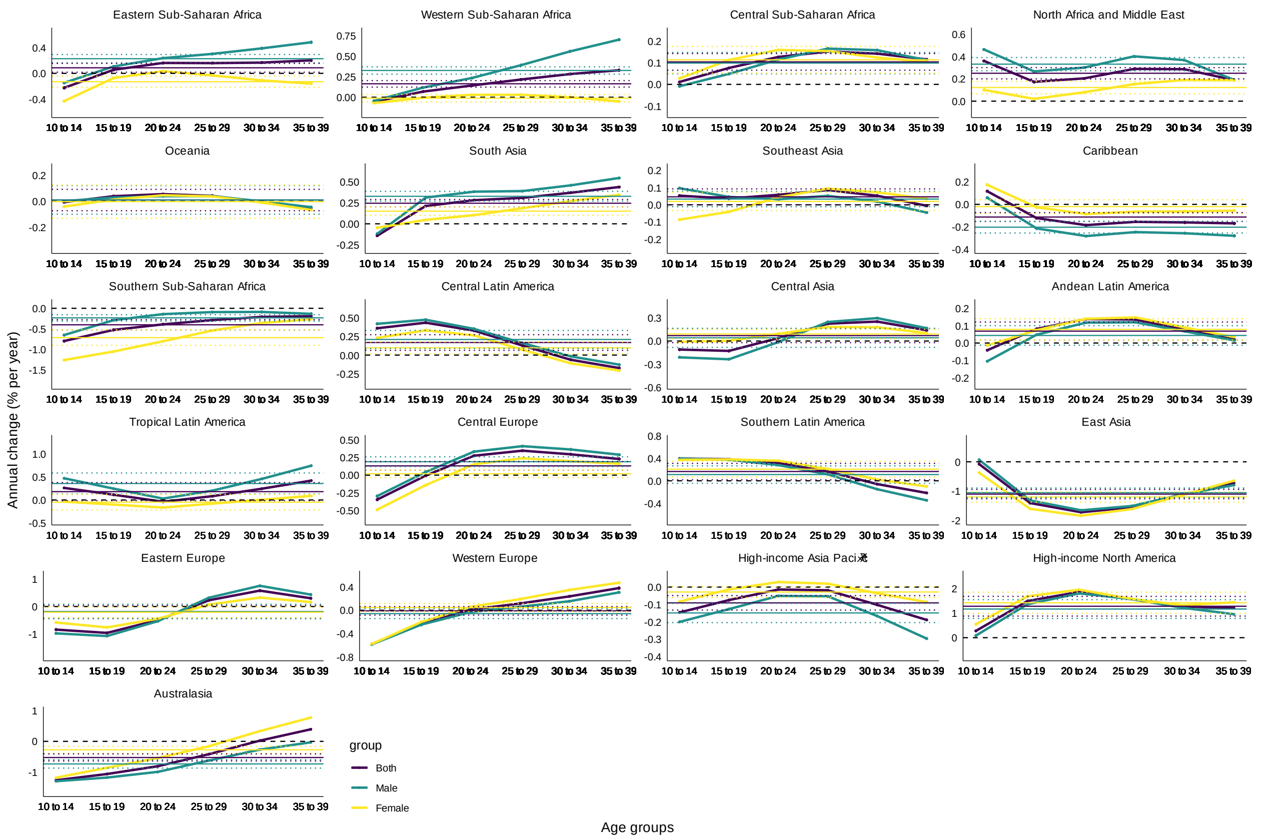

sFig. 6 Age-specific annual change of DUDs burden by regional level, sex, and year (1990-2021)

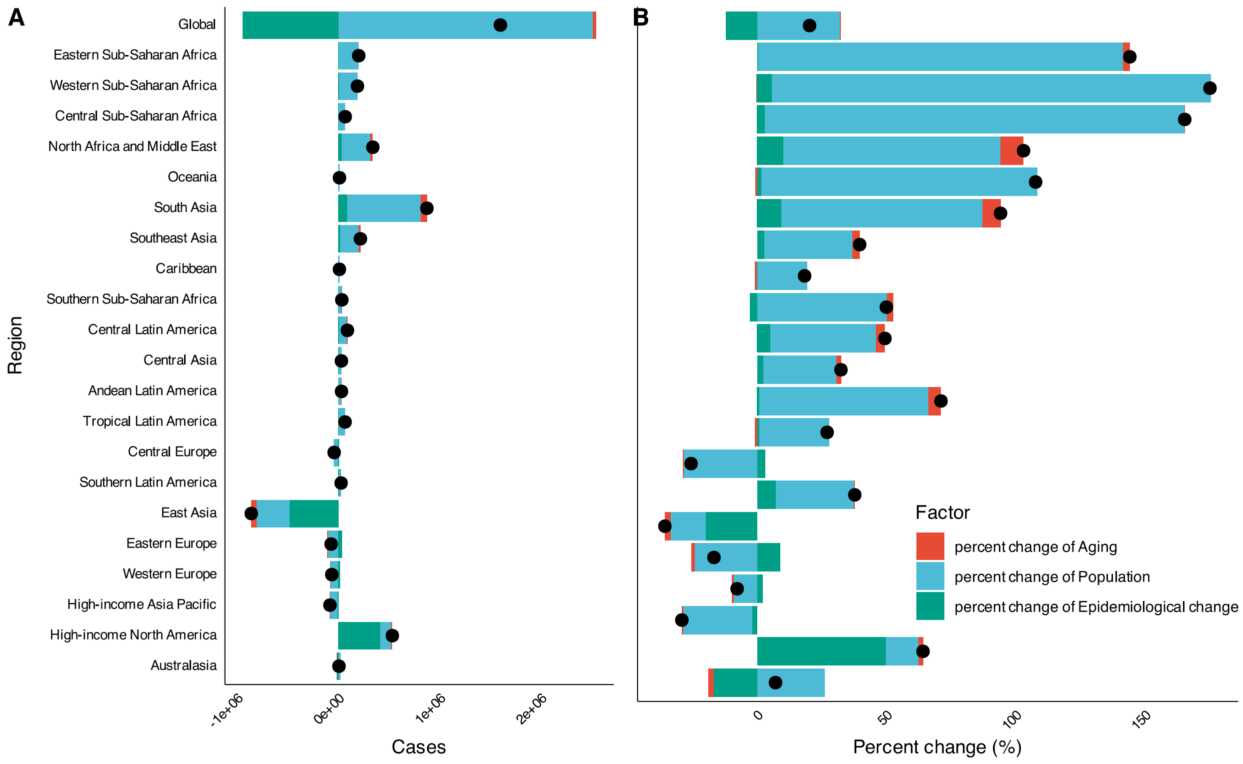

sFig. 7 Drivers factors of DUDs incidence from 1990 to 2021. A. The number of changes contributed by all 3 factors. B. The percent of change contributed by all 3 factors.

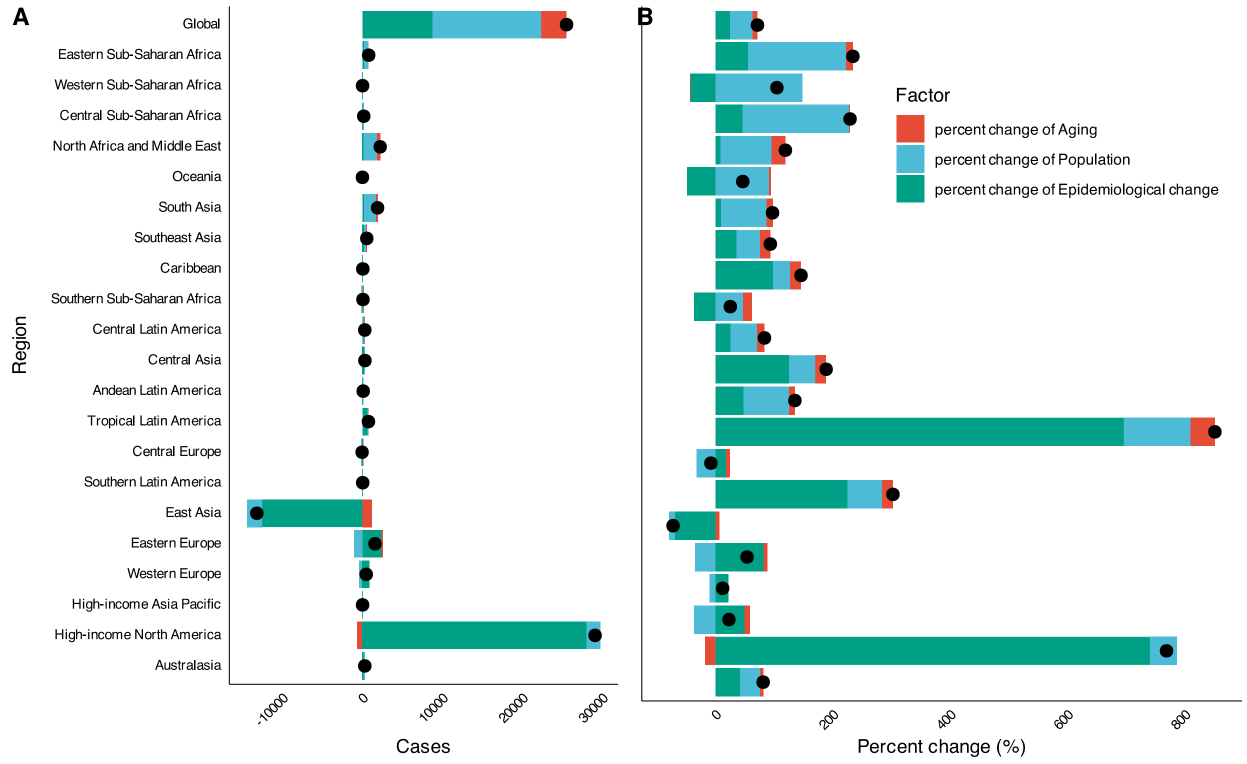

sFig. 8 Drivers factors of DUDs mortality from 1990 to 2021. A. The number of changes contributed by all 3 factors. B. The percent of change contributed by all 3 factors.

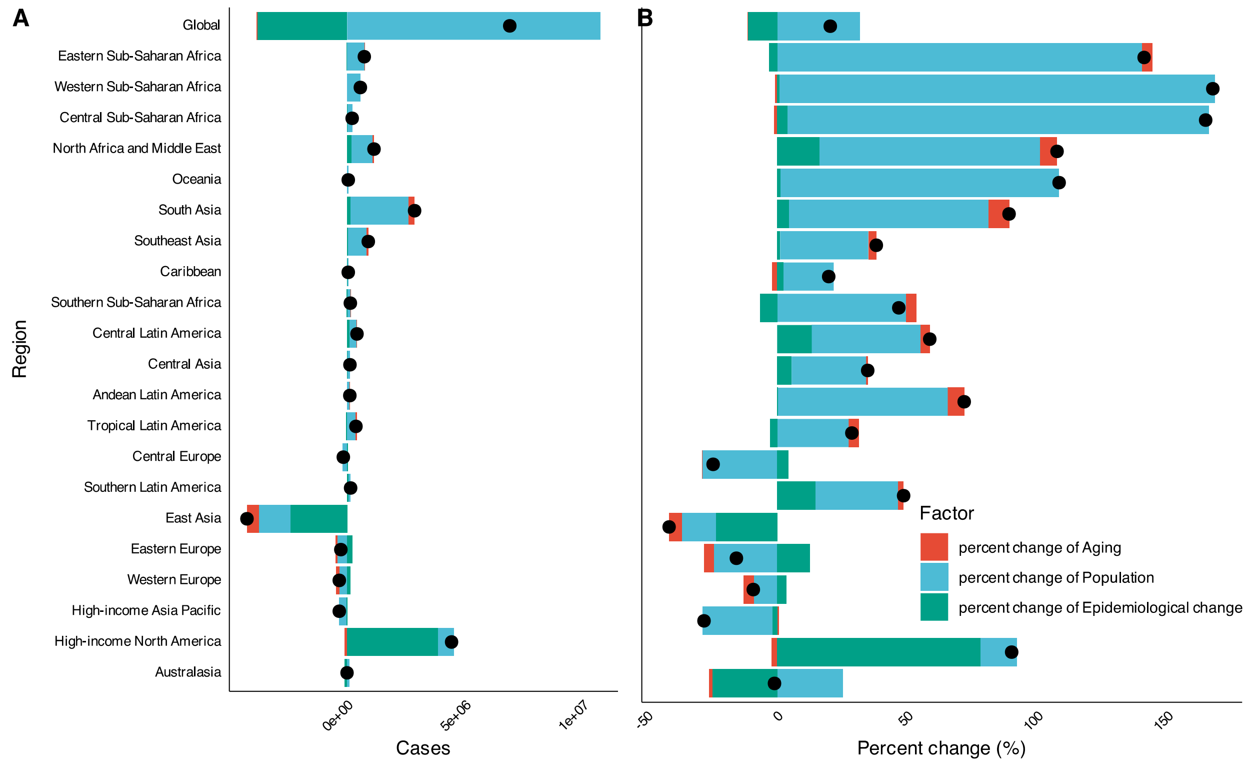

sFig. 9 Drivers factors of DUDs prevalence from 1990 to 2021. A. The number of changes contributed by all 3 factors. B. The percent of change contributed by all 3 factors.

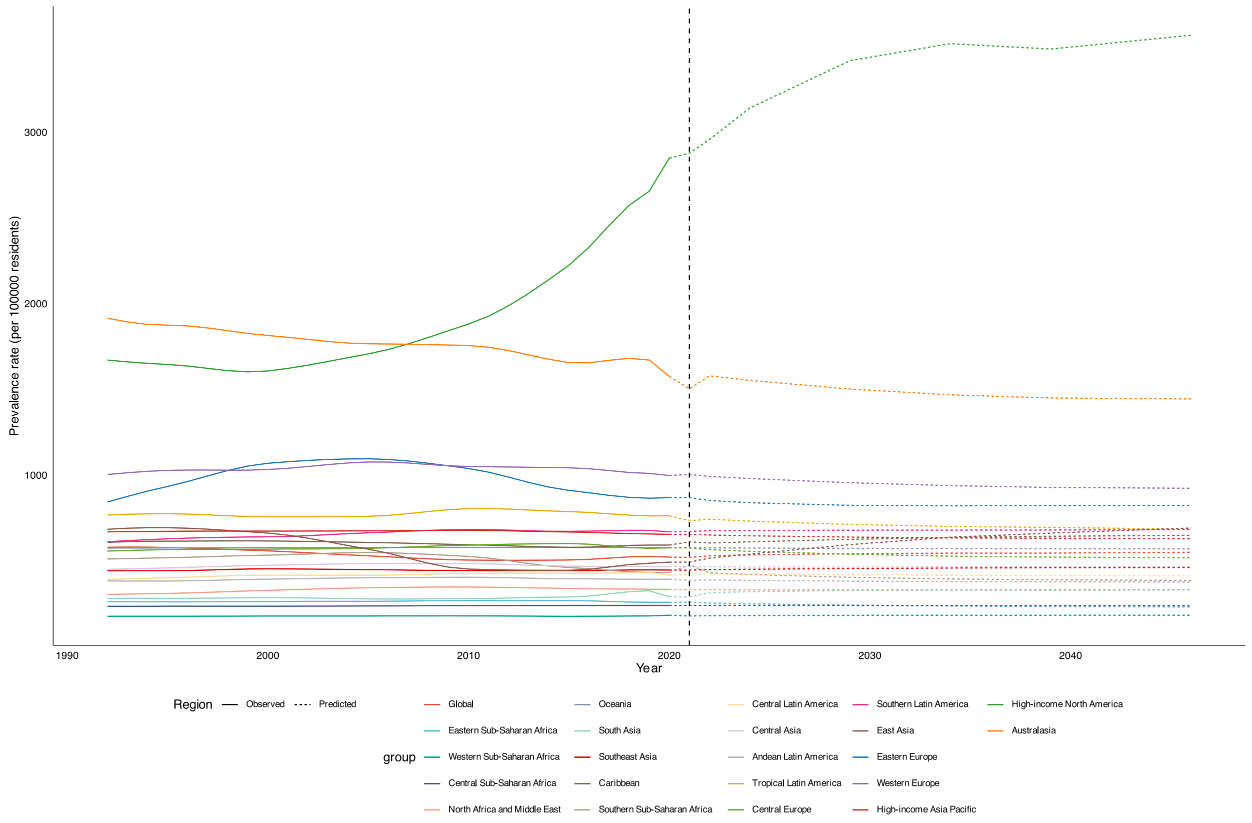

sFig. 10 Prediction of DUDs burden in the next 20 years by regions.

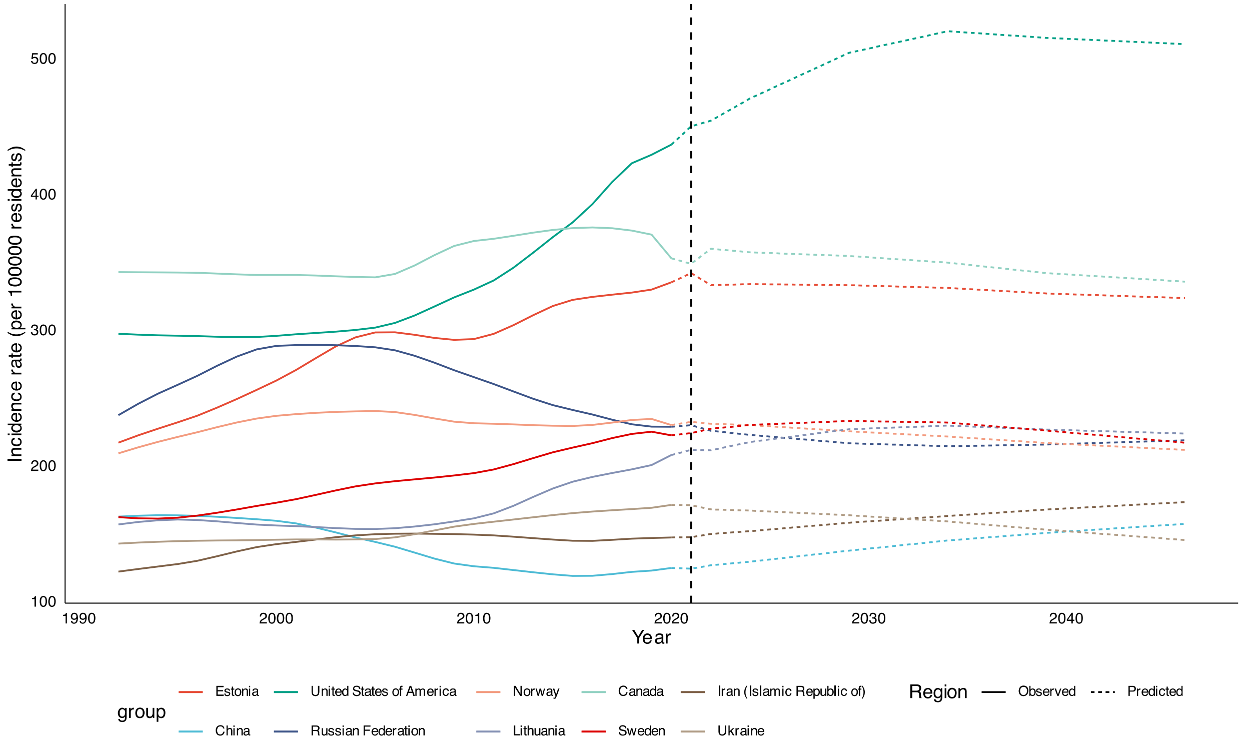

sFig. 11 Prediction of DUDs incidence rate of top 10 countries in the next 20 years.
