# Supplementary material for: Global burden on drug use disorders from 1990 to 2021 and projections to 2046
Source: Front Public Health. 2025 Jul 28;13:1550518. doi: 10.3389/fpubh.2025.1550518 (PMC12336172; doi:10.3389/fpubh.2025.1550518)
Supplement: Supplementary file 2 [file Table_1.pdf]

sTable 1 Number, crude rate, age-standardized DALYs rate for overall DUDs in 2021 and percentage change from 1990

	DALYs number		DALYs rate		Overall		Opioid		Cocaine		Amphetamine		Cannabis		Other drug	
location	Number	EAPC	Crude rate	EAPC	ASR	EAPC	ASR	EAPC	ASR	EAPC	ASR	EAPC	ASR	EAPC	ASR	EAPC
Tropical Latin America	162061.67(110807.96 - 213561.19)	-2.1(-2.44 - -1.88)	106.23(72.64 - 139.99)	-0.83(-1.17 - -0.61)	102.09(71.29 - 133.35)	-0.77(-1.03 - -0.59)	41.29(27.16 - 55.97)	0.23(0.18 - 0.26)	21.2(13.77 - 31.77)	-2.68(-3.25 - -2.19)	24.06(13.51 - 37.6)	0.11(0.04 - 0.1)	13.91(7.96 - 22.24)	0.45(0.29 - 0.49)	1.63(1.24 - 2.14)	-4.44(-4.95 - -3.94)
Global	8910603.44(7055603.29 - 10630912.05)	-1.78(-1.89 - -1.71)	167.06(132.29 - 199.32)	-0.53(-0.64 - -0.46)	166.44(132.55 - 198.4)	-0.44(-0.53 - -0.37)	103.69(81.83 - 122.75)	-0.9(-1.02 - -0.88)	10.91(7.86 - 14.84)	-0.77(-1.13 - -0.53)	29.63(19.51 - 43.52)	1.12(0.95 - 1.28)	8.63(5.1 - 13.25)	0.14(0.13 - 0.1)	13.58(11.05 - 18.14)	0.78(0.41 - 1.4)
High-income North America	1078039.6(831794.64 - 1308306.68)	-5.77(-6.05 - -5.61)	383.09(295.58 - 464.91)	-4.93(-5.22 - -4.78)	352.05(270.73 - 427.06)	-5.19(-5.47 - -5.04)	207.65(165.2 - 248.95)	-6.18(-6.29 - -6.08)	67.79(45.53 - 97.79)	-2.48(-3.13 - -2.02)	32.67(18.88 - 50.76)	-3.5(-4.54 - -2.81)	29.53(17.89 - 45.14)	0.19(0.17 - 0.17)	14.41(12.77 - 16.45)	-4.48(-4.47 - -4.5)
Central Latin America	139214.36(105074.32 - 176664.83)	-1.71(-1.8 - -1.64)	84.68(63.91 - 107.46)	-0.33(-0.43 - -0.26)	86.88(65.99 - 109.39)	-0.06(-0.14 - -0.01)	43.16(29.44 - 56.57)	0.22(0.15 - 0.22)	25.39(18.95 - 34.28)	-0.03(0 - 0)	7.43(4.46 - 11.21)	-0.53(-0.8 - -0.36)	6.43(3.92 - 9.86)	-0.68(-0.75 - -0.59)	4.48(3.84 - 5.24)	-0.78(-0.83 - -0.74)
South Asia	686910.37(536014.83 - 854810.86)	-2.53(-2.56 - -2.49)	62.82(49.02 - 78.18)	-0.87(-0.9 - -0.83)	71.11(56.1 - 87.26)	-0.33(-0.33 - -0.29)	55.72(42.26 - 68.1)	-0.33(-0.32 - -0.35)	2.76(1.34 - 4.81)	-0.17(-1.3 - 0.58)	2.02(1.33 - 3.13)	-0.54(-0.67 - -0.23)	7.59(4.48 - 12.05)	0.05(0.01 - 0.02)	3.02(2.1 - 5.66)	-1.05(-1.62 - 0.3)

	DALYs number		DALYs rate		Overall		Opioid		Cocaine		Amphetamine		Cannabis		Other drug	
location	Number	EAPC	Crude rate	EAPC	ASR	EAPC	ASR	EAPC	ASR	EAPC	ASR	EAPC	ASR	EAPC	ASR	EAPC
High-income Asia Pacific	166825.47(118067.02 - 220263.36)	0.29(0.17 - 0.4)	96.22(68.1 - 127.04)	0.51(0.39 - 0.61)	89.87(63.71 - 119.15)	-0.01(-0.07 - 0.04)	43.18(29.21 - 57.21)	-0.04(-0.18 - 0.01)	14.88(8.99 - 23.47)	0.03(0.07 - 0.04)	15.18(8.42 - 23.49)	0(-0.1 - - 0.05)	13.69(7.79 - 22.47)	0.04(0.04 - 0.01)	2.94(2.08 - 4.14)	0.1(0.04 - 0.18)
East Asia	3614386.8(2897066.28 - 4299278.97)	2.39(2.51 - 2.31)	296.88(237.96 - 353.14)	3.02(3.14 - 2.94)	268.49(217.53 - 316.18)	2.71(2.89 - 2.56)	153.51(120.43 - 181.16)	3.41(3.54 - 3.24)	2.96(2.04 - 4.13)	2.19(2.37 - 2.16)	73.34(50.41 - 104.33)	1.61(1.73 - 1.52)	4.85(2.82 - 7.75)	-0.69(-0.64 - - 0.71)	33.83(25.87 - 48.59)	3.79(3.69 - 4.35)
Central Europe	124195.04(97749.04 - 151799.02)	0(-0.08 - 0.07)	99.28(78.14 - 121.35)	-0.26(-0.34 - - 0.2)	98.28(77.04 - 120.4)	-0.47(-0.53 - - 0.42)	52.45(41.21 - 63.16)	-0.66(-0.82 - - 0.59)	7.79(5.84 - 10.86)	0.67(1.09 - 0.43)	21.57(12.6 - 33.39)	-0.7(-0.85 - - 0.63)	10.36(6.26 - 16.38)	0.12(0.07 - 0.23)	6.11(5.29 - 7.03)	-0.06(-0.05 - - 0.07)
Central Asia	92368.72(67553.27 - 118438.49)	-1.73(-1.89 - - 1.56)	133.27(97.46 - 170.88)	-0.7(-0.86 - - 0.52)	135.73(100.13 - 171.36)	-0.5(-0.64 - - 0.38)	104.53(75.93 - 132.93)	-0.38(-0.53 - - 0.27)	5.11(3.74 - 7.06)	-1.46(-1.63 - - 1.18)	16.12(9.58 - 24.82)	-0.72(-0.98 - - 0.51)	5.56(2.96 - 9.47)	-0.12(-0.05 - - 0.08)	4.41(3.46 - 5.48)	-1.49(-1.66 - - 1.34)
Eastern Europe	768334.28(614076.36 - 907539.44)	-0.26(-0.47 - - 0.2)	339.23(271.13 - 400.7)	-0.56(-0.76 - - 0.49)	323.6(257.79 - 382.26)	-0.71(-0.87 - - 0.65)	250.75(195.86 - 302.22)	-0.69(-0.9 - - 0.61)	16.25(13.63 - 19.71)	0.71(0.89 - 0.5)	28.68(18.13 - 42.77)	-1.03(-1.46 - - 0.71)	9.2(5.29 - 14.86)	-0.2(-0.1 - -0.15)	18.73(16.86 - 20.82)	-1.47(-1.5 - - 1.42)

	DALYs number		DALYs rate		Overall		Opioid		Cocaine		Amphetamine		Cannabis		Other drug	
**location**	**Number**	**EAPC**	**Crude rate**	**EAPC**	**ASR**	**EAPC**	**ASR**	**EAPC**	**ASR**	**EAPC**	**ASR**	**EAPC**	**ASR**	**EAPC**	**ASR**	**EAPC**
Eastern Sub-Saharan Africa	86968.65(66611.07 - 111945.93)	-3.23(-3.26 - -3.12)	45.58(34.91 - 58.66)	-0.69(-0.72 - -0.57)	60.83(47.07 - 78.07)	-0.29(-0.31 - -0.16)	46.11(34.98 - 59.66)	-0.36(-0.43 - -0.17)	2.45(1.32 - 4.28)	0.14(-0.31 - 0.01)	5.01(2.9 - 7.96)	-0.14(-0.17 - -0.03)	6.34(3.66 - 10.08)	0.04(0.04 - -0.01)	0.91(0.56 - 1.43)	-0.62(-0.83 - -0.49)
North Africa and Middle East	453680.83(353056.32 - 564082.79)	-2.7(-2.8 - -2.57)	133.75(104.09 - 166.3)	-0.77(-0.87 - -0.64)	148.73(117.32 - 181.45)	-0.27(-0.33 - -0.2)	118.35(89.71 - 146.66)	-0.27(-0.34 - -0.23)	8.29(5.05 - 13.21)	0.47(-0.27 - 1.13)	5.59(3.6 - 8.37)	-0.65(-0.72 - -0.38)	3.43(1.96 - 5.52)	-0.42(-0.47 - -0.4)	13.07(8.96 - 22.4)	-0.5(-1.18 - 0.63)
Western Sub-Saharan Africa	60464.33(42963.92 - 79862.2)	-3.02(-3.01 - -3.01)	31.3(22.24 - 41.35)	-0.07(-0.06 - -0.06)	37.9(27.48 - 48.72)	0.05(0.09 - 0.04)	27.72(18.96 - 36.24)	0.1(0.13 - 0.08)	1.13(0.79 - 1.6)	-0.39(-0.29 - -0.39)	4.67(2.62 - 7.53)	0.08(0.1 - 0.09)	3.56(2.02 - 5.56)	-0.06(-0.09 - -0.1)	0.82(0.47 - 1.28)	-0.52(-0.69 - -0.4)
Caribbean	33153.26(23763.25 - 42405.06)	-1.01(-1.21 - -0.97)	93.94(67.33 - 120.15)	-0.06(-0.27 - -0.02)	90.76(65.78 - 116.08)	-0.05(-0.22 - -0.03)	48.49(32.7 - 64.37)	0.56(0.48 - 0.6)	19.22(13.18 - 28.23)	-0.78(-1.02 - -0.55)	6.86(3.86 - 10.76)	-0.69(-1.06 - -0.5)	13.07(7.12 - 21.44)	-0.16(-0.14 - -0.17)	3.12(2.37 - 4.06)	-1.4(-1.69 - -1.16)
Southeast Asia	324375.96(232647.55 - 428994.35)	-1.55(-1.65 - -1.45)	69.68(49.98 - 92.16)	-0.26(-0.36 - -0.15)	68.29(50.24 - 88.66)	-0.14(-0.15 - -0.09)	30.74(23.12 - 38.19)	-0.22(-0.28 - -0.19)	1.05(0.72 - 1.44)	-0.26(-0.49 - -0.44)	26.81(15.33 - 42.23)	0(-0.08 - 0.04)	7.48(4.3 - 11.97)	-0.15(-0.16 - -0.18)	2.21(1.72 - 2.87)	-0.38(-0.17 - -0.37)
Oceania	4562.72(3240.99	-2.4(-2.43 - -2.32)	69.66(49.48 - 93.79)	0.01(-0.02 - 0.09)	71.33(51.57 - 93.2)	0.12(0.12 - 0.12)	36.62(26.83 - 47.44)	0.21(0.31 - 0.2)	1.1(0.68 - 1.76)	0.58(0.19 - 0.72)	18.32(10.13 - 29.59)	-0.02(0.0	13.07(6.78 - 22.81)	-0.03(-0.17 - 0.09)	2.22(1.56 - 3.17)	0.36(0.44 - 0.3)

	DALYs number		DALYs rate		Overall		Opioid		Cocaine		Amphetamine		Cannabis		Other drug	
location	Number	EAPC	Crude rate	EAPC	ASR	EAPC	ASR	EAPC	ASR	EAPC	ASR	EAPC	ASR	EAPC	ASR	EAPC
	- 6143.33)											1 - - 0.05)				
Australasia	79156.56(63933.22 - 93223.68)	-1.95(-2.08 - -1.88)	390.39(315.31 - 459.77)	-0.6(-0.73 - -0.53)	365.23(294.85 - 430.61)	-0.77(-0.88 - -0.73)	194.37(161.72 - 225.02)	-0.18(-0.12 - -0.21)	28.88(17.39 - 45.95)	-0.41(-0.45 - -0.35)	65.48(38.31 - 102.45)	-0.71(-1.08 - -0.44)	35.19(22.77 - 51.28)	1.73(1.71 - 1.62)	41.31(36.93 - 45.91)	-3.48(-3.45 - -3.48)
Southern Sub-Saharan Africa	82678.22(64117.82 - 100809.1)	-1.11(-1.27 - -1.01)	157.73(122.32 - 192.32)	0.26(0.1 - 0.36)	175.43(138.47 - 210.37)	0.66(0.54 - 0.71)	121.87(93.86 - 147.22)	1.02(0.87 - 1.09)	16.46(12.09 - 22.78)	-0.16(-0.09 - -0.1)	18.29(10.99 - 27.48)	0.14(-0.09 - 0.32)	7.3(4.28 - 11.46)	-0.44(-0.35 - -0.54)	11.51(9.54 - 14.07)	0.1(-0.1 - 0.32)
Southern Latin America	51692.88(35509.65 - 68800.7)	-1.34(-1.49 - -1.26)	104.35(71.68 - 138.88)	-0.34(-0.49 - -0.27)	104.52(72.1 - 139.02)	-0.17(-0.3 - -0.11)	51.92(33.12 - 69.72)	0.01(-0.28 - 0.09)	32.28(19.05 - 51.34)	-0.27(-0.32 - -0.14)	9.4(5.26 - 15.29)	-0.22(-0.39 - -0.13)	8.39(5.29 - 12.81)	-0.77(-0.99 - -0.62)	2.53(1.69 - 3.61)	-0.3(-0.48 - -0.2)
Western Europe	847230.94(685035.12 - 1005491.98)	-1.02(-1.13 - -0.94)	220.4(178.2 - 261.57)	-0.6(-0.72 - -0.53)	211.34(170.89 - 250.97)	-0.86(-0.96 - -0.8)	136.19(113.6 - 159.53)	-0.86(-0.89 - -0.84)	20.73(13.42 - 30.4)	-0.56(-0.7 - -0.46)	23.53(14.04 - 36.51)	-1(-1.39 - -0.73)	17.95(11.31 - 26.84)	0.12(0.02 - 0.16)	12.95(11.26 - 14.99)	-1.98(-2.11 - -1.84)
Central Sub-Saharan Africa	23760.59(17783.58 - 30833.37)	-3.45(-3.37 - -3.42)	43.23(32.36 - 56.1)	-0.57(-0.48 - -0.54)	52.77(39.86 - 67.88)	-0.37(-0.29 - -0.34)	39.45(28.8 - 51.84)	-0.43(-0.39 - -0.36)	1.96(1.24 - 3.24)	-0.45(-0.54 - -0.37)	5.09(2.76 - 8.08)	-0.2(-0.27 - -0.11)	5.13(2.75 - 8.5)	-0.05(-0.06 - -0.01)	1.15(0.65 - 1.69)	-0.29(-0.5 - -0.33)

	DALYs number		DALYs rate		Overall		Opioid		Cocaine		Amphetamine		Cannabis		Other drug	
location	Number	EAPC	Crude rate	EAPC	ASR	EAPC	ASR	EAPC	ASR	EAPC	ASR	EAPC	ASR	EAPC	ASR	EAPC
Andean Latin America	30542.19(22396.51 - 38683.36)	-2.38(-2.56 - -2.32)	80.39(58.95 - 101.82)	-0.61(-0.8 - -0.56)	83.02(62.22 - 104.32)	-0.32(-0.45 - -0.28)	43.69(29.09 - 59.24)	-0.18(-0.37 - -0.15)	16.74(12.96 - 21.58)	-0.79(-0.8 - -0.77)	9.17(5.14 - 14.16)	-0.59(-0.95 - -0.48)	7.07(4.09 - 11.19)	0.1(0.05 - 0.08)	6.34(5.19 - 7.61)	0.14(0.24 - -0.02)

Notes: ASR, age-standardized rate per 100000 residents; EAPC, estimated annual percent change (%); data in () indicates the uncertainty interval, it reflects the certainty of an estimate based on data availability, studies size and consistency across data sources.
