# Supplementary material for: Global burden on drug use disorders from 1990 to 2021 and projections to 2046
Source: Front Public Health. 2025 Jul 28;13:1550518. doi: 10.3389/fpubh.2025.1550518 (PMC12336172; doi:10.3389/fpubh.2025.1550518)
Supplement: Supplementary file 3 [file Table_2.pdf]

sTable 2 Number, crude rate, age-standardized incidence rate for overall DUDs in 2021 and percentage change from 1990

	Incidence number		Incidence rate		Overall		Opioid		Cocaine		Amphetamine		Cannabis		Other drug	
**location**	**Number**	**EAPC**	**Crude rate**	**EAPC**	**ASR**	**EAPC**	**ASR**	**EAPC**	**ASR**	**EAPC**	**ASR**	**EAPC**	**ASR**	**EAPC**	**ASR**	**EAPC**
Global	13609362.38(11625287.78 - 15667184.2)	0.98(1 - 1)	172.46(147.32 - 198.54)	-0.28(-0.27 - -0.27)	169.39(145.14 - 195.01)	-0.27(-0.25 - -0.26)	24.54(20.74 - 29.48)	0.16(0.19 - 0.11)	2.87(2.06 - 3.93)	-0.25(-0.11 - -0.35)	13.72(9.7 - 19.07)	-1.61(-1.58 - -1.63)	46.77(35.25 - 61.17)	-0.11(-0.1 - -0.11)	81.49(62.45 - 103.64)	-0.2(-0.16 - -0.23)
Central Europe	194938.56(163006.54 - 228660.21)	-0.41(-0.4 - -0.43)	169.12(141.42 - 198.38)	-0.15(-0.14 - -0.17)	184.24(155.27 - 214.63)	0.15(0.17 - 0.1)	16.1(13.62 - 18.96)	0.62(0.74 - 0.51)	3.08(2 - 4.57)	0.15(0.17 - 0.16)	23.83(16.19 - 33.54)	0.44(0.51 - 0.37)	55.12(42.33 - 71.28)	-0.2(-0.08 - -0.31)	86.12(63.91 - 111.03)	0.23(0.24 - 0.19)
East Asia	2553499.27(2134777.93 - 3029328.25)	-0.53(-0.57 - -0.46)	173.38(144.95 - 205.69)	-1.14(-1.18 - -1.07)	173.93(146.09 - 204.63)	-0.73(-0.76 - -0.69)	16.71(13.88 - 20.27)	-1.9(-1.96 - -1.87)	0.72(0.46 - 1.05)	-0.65(-0.75 - -0.62)	32.31(22.4 - 45.25)	-1.44(-1.49 - -1.36)	36.16(26.09 - 49.11)	0.64(0.55 - 0.61)	88.03(66.19 - 112.56)	-0.62(-0.65 - -0.6)
Oceania	24811.51(20002.28 - 30730.38)	2.52(2.59 - 2.42)	178.15(143.62 - 220.64)	0.06(0.13 - -0.04)	173.25(141.6 - 212)	0.02(0.05 - 0)	12.49(10.2 - 15.23)	0.11(0.1 - 0.09)	0.28(0.16 - 0.42)	-0.2(-0.39 - -0.12)	17.24(11.45 - 25)	0(0.03 - 0)	72.23(47.14 - 102.46)	0.03(0.04 - -0.05)	71.01(54.14 - 90.31)	0(0.07 - -0.05)
Central Asia	165880.08(140144.05 - 194128.96)	1.26(1.27 - 1.21)	173.14(146.27 - 202.62)	0.21(0.22 - 0.16)	169.72(143.57 - 197.09)	0.08(0.07 - 0.03)	36.68(30.96 - 43.7)	0.03(0.14 - -0.05)	2.1(1.42 - 2.99)	0.24(0.38 - 0.13)	16.61(11.63 - 23.13)	0.18(0.24 - 0.14)	33.79(22.62 - 50.51)	0.1(0.11 - 0.08)	80.53(60 - 102.81)	0.06(0.09 - 0.03)
Southeast Asia	1049717.45(869353.95 - 1231732.54)	1.51(1.53 - 1.49)	150.32(124.49 - 176.39)	0.19(0.21 - 0.17)	141.48(116.93 - 166.01)	0.1(0.1 - 0.08)	9.36(7.79 - 11.23)	0.05(0.09 - 0)	0.24(0.14 - 0.36)	-0.42(-0.48 - -0.37)	22.65(15.16 - 32.8)	-0.1(-0.03 - -0.13)	46.3(33.74 - 63.67)	0.15(0.16 - 0.15)	62.94(46.97 - 81.27)	0.14(0.13 - 0.11)
High-income Asia Pacific	324897.1(268440.1 - 387744.63)	-0.57(-0.55 - -0.61)	175.2(144.75 - 209.09)	-0.79(-0.77 - -0.82)	204.38(168.19 - 247.27)	-0.07(-0.05 - -0.08)	14.92(12.05 - 18.42)	-0.05(0 - -0.08)	6.5(4.43 - 9.5)	-0.13(-0.1 - -0.06)	15.15(10.11 - 21.23)	-0.07(-0.06 - -0.12)	78.59(55.32 - 109.81)	-0.04(0.02 - -0.03)	89.22(64.91 - 117.3)	-0.09(-0.09 - -0.1)

	Incidence number		Incidence rate		Overall		Opioid		Cocaine		Amphetamine		Cannabis		Other drug	
**location**	**Number**	**EAPC**	**Crude rate**	**EAPC**	**ASR**	**EAPC**	**ASR**	**EAPC**	**ASR**	**EAPC**	**ASR**	**EAPC**	**ASR**	**EAPC**	**ASR**	**EAPC**
Central Latin America	384443.57(323943.49 - 446656.3)	1.72(1.72 - 1.7)	151.95(128.04 - 176.54)	0.32(0.31 - 0.29)	144.04(121.38 - 167.38)	0.06(0.09 - 0.03)	15.25(12.19 - 18.85)	-0.17(-0.15 - -0.2)	7.56(4.83 - 11.58)	-0.14(-0.12 - -0.16)	6.75(4.59 - 9.38)	0.01(-0.01 - -0.04)	42.34(32.65 - 54.46)	0.52(0.66 - 0.44)	72.15(54.14 - 93.22)	-0.11(-0.12 - -0.1)
Western Europe	1158666.62(1007512.77 - 1338089.27)	0.18(0.25 - 0.17)	264.91(230.35 - 305.93)	-0.24(-0.17 - -0.25)	302(262.87 - 348.16)	0.13(0.18 - 0.11)	24.07(20.69 - 28.09)	0.35(0.4 - 0.28)	9.53(6.2 - 14.54)	0.17(0.15 - 0.27)	25.31(17.31 - 35.24)	0.27(0.3 - 0.26)	96.2(76.54 - 119.49)	-0.28(-0.29 - -0.28)	146.89(114.29 - 184.79)	0.36(0.5 - 0.27)
Andean Latin America	102256.01(86119.97 - 118551.78)	2.11(2.11 - 2.09)	154.62(130.22 - 179.26)	0.3(0.3 - 0.29)	147.25(123.64 - 171.11)	0.06(0.08 - 0.03)	17.39(13.8 - 21.67)	0.07(0.13 - 0.05)	5.27(3.32 - 8.08)	0.16(0.24 - 0.08)	9.29(6.33 - 13.13)	0.18(0.26 - 0.11)	39.76(29.1 - 52.9)	-0.07(-0.1 - -0.05)	75.53(55.9 - 97.52)	0.1(0.07 - 0.1)
North Africa and Middle East	950174.79(796661.95 - 1121367.53)	2.68(2.69 - 2.67)	152.52(127.88 - 179.99)	0.69(0.69 - 0.68)	143.52(120.87 - 169.07)	0.22(0.23 - 0.22)	37.82(31.5 - 45.62)	0.27(0.37 - 0.19)	1.62(1.07 - 2.34)	-0.12(-0.08 - -0.13)	4.75(3.3 - 6.67)	0.21(0.29 - 0.2)	22.73(16.1 - 31.39)	0.34(0.35 - 0.33)	76.6(57.6 - 99.3)	0.17(0.21 - 0.14)
Eastern Europe	529776.02(457123.96 - 607119.51)	-0.36(-0.33 - -0.36)	256.23(221.09 - 293.64)	-0.07(-0.04 - -0.07)	275.72(238.8 - 312.9)	0.16(0.19 - 0.13)	73.32(61.9 - 87.26)	0.17(0.22 - 0.13)	4.35(3.13 - 6.05)	0.38(0.52 - 0.27)	26.25(18.74 - 35.39)	0.17(0.25 - 0.08)	55.89(37.85 - 79.13)	0.19(0.11 - 0.21)	115.92(87.05 - 146.22)	0.13(0.13 - 0.09)
Australasia	123722.95(106674.83 - 141155.62)	0.68(0.64 - 0.6)	399.6(344.54 - 455.91)	-0.69(-0.73 - -0.76)	425.48(369.38 - 483.04)	-0.37(-0.38 - -0.45)	44.87(38.68 - 51.99)	0.11(0.1 - 0.12)	12.43(8.34 - 18.59)	0.19(0.17 - 0.21)	55.68(38.08 - 78.43)	-0.16(-0.09 - -0.15)	114.65(90.13 - 144.62)	-1.5(-1.55 - -1.57)	197.85(155.75 - 246.44)	0.3(0.43 - 0.15)
Southern Latin America	135147.98(114721.72 - 157009.46)	1.23(1.26 - 1.19)	199.64(169.47 - 231.94)	0.22(0.25 - 0.17)	196.13(167.54 - 227.34)	0.17(0.22 - 0.12)	17.75(14.11 - 22.1)	-0.02(0.04 - -0.02)	14.82(9.74 - 22.82)	0.13(0.17 - 0.17)	10.73(7.23 - 15.12)	0.03(0.05 - 0.04)	59.35(48.47 - 72.21)	0.57(0.7 - 0.45)	93.48(68.39 - 121.15)	0(-0.01 - -0.07)

	Incidence number		Incidence rate		Overall		Opioid		Cocaine		Amphetamine		Cannabis		Other drug	
**location**	**Number**	**EAPC**	**Crude rate**	**EAPC**	**ASR**	**EAPC**	**ASR**	**EAPC**	**ASR**	**EAPC**	**ASR**	**EAPC**	**ASR**	**EAPC**	**ASR**	**EAPC**
High-income North America	1713708(1503719.89 - 1941995.9)	1.68(1.75 - 1.62)	462.95(406.22 - 524.62)	0.79(0.86 - 0.72)	520.07(454.13 - 592.82)	1.13(1.21 - 1.07)	144.24(120.13 - 174.95)	5.18(5.22 - 5.08)	23.87(17.69 - 33.93)	0.26(0.58 - 0.16)	42(29.89 - 56.72)	0.93(1.31 - 0.64)	151.3(114.29 - 196.4)	-0.17(-0.17 - -0.14)	158.67(122.5 - 205.36)	0.81(0.92 - 0.81)
Caribbean	86450.43(70519.18 - 104743.81)	0.84(0.83 - 0.81)	182.16(148.59 - 220.71)	-0.12(-0.13 - -0.15)	180.09(147.21 - 220.4)	-0.01(-0.05 - 0.03)	15.61(12.48 - 19.26)	-0.47(-0.47 - -0.48)	7.82(4.84 - 11.94)	0.07(-0.02 - 0.04)	6.95(4.79 - 9.85)	0.13(0.15 - 0.17)	74.97(49.43 - 108.44)	0.19(0.08 - 0.24)	74.74(56.94 - 95.65)	-0.12(-0.13 - -0.13)
Tropical Latin America	422225.68(359194.35 - 486314.89)	1.27(1.32 - 1.1)	185.57(157.87 - 213.74)	-0.03(0.02 - -0.19)	180.4(153.43 - 207.8)	0.1(0.14 - -0.02)	15.82(12.43 - 19.74)	-0.25(-0.23 - -0.28)	12.04(8.21 - 18.04)	1.38(1.58 - 1.34)	19.4(12.91 - 27.72)	-0.09(-0.11 - -0.12)	64.81(48.52 - 84.57)	-0.38(-0.21 - -0.59)	68.33(51.33 - 88.43)	0.6(0.67 - 0.57)
Eastern Sub-Saharan Africa	417961.56(340921.71 - 513395.63)	2.99(3.03 - 2.98)	98.09(80.01 - 120.49)	0.36(0.4 - 0.35)	101.09(83.77 - 119.6)	0.07(0.1 - 0.06)	10.77(8.94 - 12.99)	0.01(0.06 - -0.04)	0.48(0.34 - 0.68)	0.43(0.54 - 0.33)	4.98(3.36 - 7)	0(-0.02 - -0.02)	36.35(25.69 - 50.62)	-0.03(-0.04 - 0.02)	48.51(36.55 - 62.36)	0.17(0.2 - 0.1)
Central Sub-Saharan Africa	138352.33(114528.91 - 166973.53)	3.29(3.33 - 3.27)	101.04(83.64 - 121.94)	0.29(0.33 - 0.27)	110.05(91.77 - 129.82)	0.07(0.08 - 0.06)	12.49(10.24 - 15.37)	0.29(0.34 - 0.28)	0.8(0.54 - 1.14)	0.34(0.43 - 0.32)	5.31(3.64 - 7.43)	0.05(0.12 - -0.01)	30.72(21.35 - 43.62)	0.01(0.01 - 0.01)	60.73(46.06 - 77.73)	0.06(0.1 - 0.09)
South Asia	2597580.43(2168940.58 - 3042955)	2.41(2.45 - 2.35)	140.67(117.46 - 164.79)	0.7(0.74 - 0.64)	131.41(109.78 - 153.28)	0.27(0.27 - 0.25)	18.9(15.73 - 23.12)	0.4(0.41 - 0.36)	0.37(0.24 - 0.54)	-0.06(-0.01 - -0.11)	1.73(1.21 - 2.43)	0.13(0.16 - 0.11)	44.47(32.43 - 59.54)	-0.05(-0.03 - -0.05)	65.94(49.97 - 84.78)	0.46(0.48 - 0.48)
Western Sub-Saharan Africa	398797.57(333659.12 - 469311.07)	3.38(3.42 - 3.35)	81.42(68.12 - 95.81)	0.32(0.37 - 0.29)	94.68(79.6 - 111.36)	0.22(0.3 - 0.16)	10.88(8.95 - 13.31)	-0.05(0.02 - -0.08)	0.7(0.53 - 0.9)	0.7(0.84 - 0.55)	4.66(3.16 - 6.6)	-0.09(-0.1 - -0.08)	21.72(15.81 - 29.29)	0.05(0.06 - 0.03)	56.73(43.19 - 71.98)	0.36(0.44 - 0.27)

	Incidence number		Incidence rate		Overall		Opioid		Cocaine		Amphetamine		Cannabis		Other drug	
location	Number	EAPC	Crude rate	EAPC	ASR	EAPC	ASR	EAPC	ASR	EAPC	ASR	EAPC	ASR	EAPC	ASR	EAPC
Southern Sub-Saharan Africa	136354.46(115591.92 - 157588.06)	1.64(1.58 - 1.59)	169.8(143.94 - 196.24)	0.25(0.19 - 0.21)	161.51(137.31 - 186.47)	0(-0.05 - 0.02)	23.31(19.68 - 28.1)	-1.11(-1.06 - -1.11)	5.51(3.89 - 7.67)	0.23(0.29 - 0.21)	11.74(8.21 - 16.33)	-0.24(-0.21 - -0.25)	46.14(32.43 - 63.24)	0.39(0.29 - 0.48)	74.81(57.43 - 93.96)	0.22(0.28 - 0.18)

Notes: ASR, age-standardized rate per 100000 residents; EAPC, estimated annual percent change (%); data in () indicates the uncertainty interval, it reflects the certainty of an estimate based on data availability, studies size and consistency across data sources.
