# Supplementary material for: Global burden on drug use disorders from 1990 to 2021 and projections to 2046
Source: Front Public Health. 2025 Jul 28;13:1550518. doi: 10.3389/fpubh.2025.1550518 (PMC12336172; doi:10.3389/fpubh.2025.1550518)
Supplement: Supplementary file 4 [file Table_3.pdf]

sTable 3 Number, crude rate, age-standardized death rate for overall DUDs in 2021 and percentage change from 1990

	Death number		Death rate		Overall		Opioid		Cocaine		Amphetamine		Other drug	
**location**	**Number**	**EAPC**	**Crude rate**	**EAPC**	**ASR**	**EAPC**	**ASR**	**EAPC**	**ASR**	**EAPC**	**ASR**	**EAPC**	**ASR**	**EAPC**
Southern Sub-Saharan Africa	931.89(848.88 - 1039.24)	1.97(2.2 - 1.97)	1.16(1.06 - 1.29)	0.58(0.8 - 0.57)	1.32(1.2 - 1.45)	0.02(0.29 - 0)	0.88(0.78 - 0.99)	-0.15(0.02 - -0.15)	0.14(0.11 - 0.17)	0.03(0.17 - -0.11)	0.07(0.05 - 0.08)	2.33(2.3 - 2.02)	0.23(0.2 - 0.27)	0.21(0.41 - -0.02)
Central Asia	888.31(744.25 - 1037.8)	3.58(3.46 - 3.64)	0.93(0.78 - 1.08)	2.5(2.39 - 2.56)	0.92(0.78 - 1.08)	1.95(1.85 - 2)	0.63(0.52 - 0.74)	1.66(1.6 - 1.65)	0.1(0.07 - 0.12)	2.4(2.45 - 2.17)	0.07(0.06 - 0.09)	5.15(5.66 - 4.05)	0.12(0.1 - 0.14)	1.99(2.15 - 1.78)
Global	137277.92(129268.62 - 146181.36)	2.61(2.66 - 2.55)	1.74(1.64 - 1.85)	1.32(1.37 - 1.27)	1.65(1.55 - 1.75)	0.87(0.92 - 0.81)	1.19(1.12 - 1.29)	1.07(1.23 - 1.08)	0.15(0.14 - 0.17)	2.38(2.67 - 2.07)	0.12(0.11 - 0.13)	0.8(1.01 - 0.56)	0.18(0.17 - 0.2)	-0.8(-0.35 - -1.65)
High-income Asia Pacific	420.93(389.96 - 448.21)	2.34(2.25 - 2.38)	0.23(0.21 - 0.24)	2.12(2.03 - 2.16)	0.17(0.16 - 0.18)	1.58(1.56 - 1.61)	0.12(0.11 - 0.13)	1.71(1.7 - 1.7)	0.02(0.02 - 0.02)	1.8(2.14 - 1.66)	0.01(0.01 - 0.01)	4.55(4.54 - 4.55)	0.02(0.02 - 0.02)	0.14(0.2 - -0.03)
Southern Latin America	155.64(138.57 - 175.81)	4.98(4.9 - 5.11)	0.23(0.2 - 0.26)	3.93(3.85 - 4.06)	0.2(0.18 - 0.23)	3.27(3.18 - 3.42)	0.14(0.12 - 0.16)	3.44(3.34 - 3.59)	0.03(0.02 - 0.03)	3.64(3.5 - 3.79)	0.01(0.01 - 0.01)	6.6(6.21 - 6.48)	0.02(0.02 - 0.03)	1.34(1.09 - 1.49)
Central Europe	975.63(901.55 - 1058.49)	0.58(0.63 - 0.55)	0.85(0.78 - 0.92)	0.85(0.9 - 0.82)	0.72(0.67 - 0.78)	0.52(0.57 - 0.48)	0.56(0.52 - 0.61)	0.72(0.79 - 0.65)	0.03(0.02 - 0.03)	-2.52(-2.53 - -2.42)	0.05(0.04 - 0.05)	2.94(2.88 - 2.78)	0.09(0.08 - 0.09)	-0.09(-0.11 - -0.08)

	Death number		Death rate		Overall		Opioid		Cocaine		Amphetamine		Other drug	
**location**	**Number**	**EAPC**	**Crude rate**	**EAPC**	**ASR**	**EAPC**	**ASR**	**EAPC**	**ASR**	**EAPC**	**ASR**	**EAPC**	**ASR**	**EAPC**
South Asia	10802.93(9459.56 - 12215.64)	2.92(2.97 - 2.87)	0.59(0.51 - 0.66)	1.2(1.24 - 1.15)	0.64(0.56 - 0.72)	0.3(0.37 - 0.24)	0.48(0.4 - 0.55)	0.09(-0.02 - 0.13)	0.06(0.04 - 0.09)	0.32(1.8 - -0.49)	0.02(0.01 - 0.02)	1.51(1.86 - 1.01)	0.09(0.07 - 0.11)	1.45(2.46 - -0.2)
Caribbean	202.43(171.07 - 235.57)	3.61(3.43 - 3.81)	0.43(0.36 - 0.5)	2.63(2.45 - 2.82)	0.4(0.34 - 0.47)	2.1(1.92 - 2.3)	0.09(0.07 - 0.11)	0.81(0.43 - 1.1)	0.2(0.16 - 0.24)	2.26(2.12 - 2.31)	0.04(0.03 - 0.04)	6(5.62 - 5.76)	0.07(0.06 - 0.09)	2.58(2.51 - 2.7)
Eastern Sub-Saharan Africa	1919.88(1290.83 - 2497.21)	3.82(3.83 - 3.48)	0.45(0.3 - 0.59)	1.17(1.17 - 0.83)	0.67(0.46 - 0.86)	0.35(0.44 - 0.01)	0.62(0.43 - 0.79)	0.36(0.4 - 0.08)	0.03(0.01 - 0.07)	-0.51(-0.09 - 0.22)	0.01(0.01 - 0.02)	2.21(2.7 - 2.25)	0.01(0.01 - 0.02)	1.51(2.61 - 0.1)
Western Europe	11741.86(11152.34 - 12283.42)	2.5(2.42 - 2.55)	2.68(2.55 - 2.81)	2.07(1.99 - 2.12)	2.28(2.19 - 2.37)	1.78(1.73 - 1.81)	1.67(1.59 - 1.74)	1.47(1.41 - 1.51)	0.11(0.11 - 0.12)	1.9(1.88 - 1.96)	0.14(0.13 - 0.15)	4.96(4.79 - 5.02)	0.36(0.34 - 0.38)	2.56(2.51 - 2.62)
Andean Latin America	346.94(284 - 426.4)	3.65(3.5 - 3.88)	0.52(0.43 - 0.64)	1.81(1.67 - 2.04)	0.52(0.43 - 0.64)	1.19(1.01 - 1.42)	0.12(0.09 - 0.16)	1.45(1.38 - 1.58)	0.26(0.21 - 0.32)	1.34(1.21 - 1.37)	0.04(0.03 - 0.05)	4.2(4.07 - 4.35)	0.1(0.08 - 0.13)	0(-0.15 - 0.17)
East Asia	11987.35(9783.26 - 14384.91)	-3.1(-3.31 - -2.95)	0.81(0.66 - 0.98)	-3.69(-3.9 - -3.55)	0.69(0.57 - 0.83)	-4.28(-4.47 - -4.15)	0.33(0.26 - 0.4)	-5.13(-5.13 - -4.99)	0.01(0.01 - 0.02)	-3.76(-4.21 - -3.87)	0.17(0.14 - 0.21)	-1.94(-2.02 - -1.98)	0.18(0.14 - 0.22)	-4.07(-4.12 - -4.59)

	Death number		Death rate		Overall		Opioid		Cocaine		Amphetamine		Other drug	
**location**	**Number**	**EAPC**	**Crude rate**	**EAPC**	**ASR**	**EAPC**	**ASR**	**EAPC**	**ASR**	**EAPC**	**ASR**	**EAPC**	**ASR**	**EAPC**
Eastern Europe	7924(7229.05 - 8651.16)	0.93(0.87 - 0.98)	3.83(3.5 - 4.18)	1.23(1.17 - 1.28)	3.41(3.11 - 3.73)	1.16(1.09 - 1.21)	2.55(2.33 - 2.81)	1.17(1.13 - 1.22)	0.12(0.11 - 0.13)	-1.82(-1.69 - -1.81)	0.22(0.2 - 0.25)	4.02(3.95 - 3.58)	0.51(0.46 - 0.56)	1.43(1.39 - 1.47)
North Africa and Middle East	7579.37(6665.44 - 8629.18)	2.98(2.96 - 2.87)	1.22(1.07 - 1.39)	0.98(0.96 - 0.87)	1.24(1.1 - 1.42)	0.12(0.14 - 0.05)	0.81(0.68 - 0.93)	0.04(0.14 - - 0.08)	0.11(0.08 - 0.14)	- 0.8(0.36 - -1.73)	0.04(0.03 - 0.06)	1.76(1.9 - 1.19)	0.28(0.24 - 0.34)	0.62(1.41 - - 0.65)
Southeast Asia	2336.48(1944.56 - 2865.15)	2.88(2.77 - 2.97)	0.33(0.28 - 0.41)	1.54(1.43 - 1.63)	0.34(0.29 - 0.42)	0.68(0.54 - 0.81)	0.25(0.2 - 0.3)	0.48(0.29 - 0.58)	0.02(0.02 - 0.03)	0.48(0.96 - 0.63)	0.03(0.02 - 0.05)	3.49(3.03 - 3.39)	0.04(0.03 - 0.06)	0.51(-0.16 - 0.77)
Central Latin America	1136.99(1005.81 - 1286.83)	2.58(2.32 - 2.83)	0.45(0.4 - 0.51)	1.17(0.9 - 1.41)	0.43(0.38 - 0.48)	0.49(0.23 - 0.74)	0.09(0.08 - 0.1)	-0.41(-0.68 - - 0.12)	0.21(0.19 - 0.24)	0.29(0.04 - 0.51)	0.04(0.03 - 0.05)	3.7(3.28 - 3.46)	0.08(0.07 - 0.09)	1.22(1.03 - 1.38)
Western Sub-Saharan Africa	129.22(86.46 - 167.79)	1.79(1.6 - 1.78)	0.03(0.02 - 0.03)	-1.22(-1.4 - - 1.23)	0.05(0.04 - 0.07)	-1.01(-0.9 - - 1.06)	0.05(0.04 - 0.07)	-1.05(-0.94 - - 1.1)	0(0 - 0)	-0.71(-1.07 - - 1.04)	0(0 - 0)	2.33(2.41 - 1.33)	0(0 - 0)	2.44(2.44 - 1.68)
Central Sub-Saharan Africa	389.62(223.82 - 595.06)	3.91(3.82 - 3.95)	0.28(0.16 - 0.43)	0.89(0.8 - 0.94)	0.38(0.22 - 0.58)	0.58(0.47 - 0.58)	0.35(0.2 - 0.53)	0.54(0.44 - 0.61)	0.02(0 - 0.05)	0.61(0.44 - 0.55)	0.01(0 - 0.01)	3.08(3.48 - 1.93)	0(0 - 0.01)	1.3(1.6 - 0.52)

	Death number		Death rate		Overall		Opioid		Cocaine		Amphetamine		Other drug	
**location**	**Number**	**EAPC**	**Crude rate**	**EAPC**	**ASR**	**EAPC**	**ASR**	**EAPC**	**ASR**	**EAPC**	**ASR**	**EAPC**	**ASR**	**EAPC**
High-income North America	74450.77(67591.35 - 82621.99)	8.39(8.24 - 8.56)	20.11(18.26 - 22.32)	7.44(7.29 - 7.6)	18.42(16.81 - 20.33)	7.55(7.42 - 7.7)	14.5(12.92 - 16.3)	7.76(7.58 - 7.95)	1.75(1.57 - 2.11)	6.72(6.54 - 7.17)	1.15(1.03 - 1.39)	9.75(9.55 - 10.2)	1.02(0.91 - 1.19)	5.39(5.14 - 5.75)
Australasia	1521.23(1361.82 - 1686.29)	3.82(3.69 - 3.94)	4.91(4.4 - 5.45)	2.41(2.28 - 2.53)	4.41(3.94 - 4.89)	2.31(2.17 - 2.43)	1.74(1.51 - 1.99)	0.44(0.24 - 0.63)	0.04(0.03 - 0.05)	6.91(6.78 - 7.1)	0.27(0.23 - 0.31)	8.53(8.34 - 8.56)	2.36(2.11 - 2.64)	4.35(4.27 - 4.46)
Tropical Latin America	1417.52(1327.08 - 1528.98)	8.02(7.98 - 8.09)	0.62(0.58 - 0.67)	6.63(6.6 - 6.71)	0.57(0.54 - 0.62)	6.08(6.04 - 6.17)	0.03(0.03 - 0.04)	4.23(4.13 - 4.32)	0.42(0.4 - 0.46)	6.21(6.15 - 6.3)	0.01(0.01 - 0.01)	3.88(3.51 - 3.99)	0.11(0.1 - 0.12)	6.75(6.69 - 6.76)
Oceania	18.94(13.73 - 26.02)	1.52(1.95 - 1.4)	0.14(0.1 - 0.19)	-0.92(-0.5 - -1.04)	0.16(0.12 - 0.22)	-1.12(-0.7 - -1.21)	0.13(0.09 - 0.17)	-1.3(-0.96 - -1.29)	0.01(0.01 - 0.02)	-0.65(-0.16 - -0.99)	0.01(0.01 - 0.02)	1.28(1.39 - 1.26)	0.02(0.01 - 0.03)	-0.94(-0.88 - -1.12)

Notes: ASR, age-standardized rate per 100000 residents; EAPC, estimated annual percent change (%); data in () indicates the uncertainty interval, it reflects the certainty of an estimate based on data availability, studies size and consistency across data sources.
